# Importance of Matrix Cues on Intervertebral Disc Development, Degeneration, and Regeneration

**DOI:** 10.3390/ijms23136915

**Published:** 2022-06-21

**Authors:** Matthew J. Kibble, Marco Domingos, Judith A. Hoyland, Stephen M. Richardson

**Affiliations:** 1 Division of Cell Matrix Biology and Regenerative Medicine, School of Biological Sciences, Faculty of Biology, Medicine and Health, University of Manchester, Oxford Road, Manchester M13 9PT, UK; matthew.kibble@postgrad.manchester.ac.uk (M.J.K.); marco.domingos@manchester.ac.uk (M.D.); judith.a.hoyland@manchester.ac.uk (J.A.H.); 2 Department of Mechanical Aerospace and Civil Engineering, School of Engineering, Faculty of Science and Engineering & Henry Royce Institute, University of Manchester, Oxford Road, Manchester M13 9PL, UK

**Keywords:** intervertebral, matrix, cues, biomimetic, laminin, bioprinting, regeneration

## Abstract

Back pain is one of the leading causes of disability worldwide and is frequently caused by degeneration of the intervertebral discs. The discs’ development, homeostasis, and degeneration are driven by a complex series of biochemical and physical extracellular matrix cues produced by and transmitted to native cells. Thus, understanding the roles of different cues is essential for designing effective cellular and regenerative therapies. Omics technologies have helped identify many new matrix cues; however, comparatively few matrix molecules have thus far been incorporated into tissue engineered models. These include collagen type I and type II, laminins, glycosaminoglycans, and their biomimetic analogues. Modern biofabrication techniques, such as 3D bioprinting, are also enabling the spatial patterning of matrix molecules and growth factors to direct regional effects. These techniques should now be applied to biochemically, physically, and structurally relevant disc models incorporating disc and stem cells to investigate the drivers of healthy cell phenotype and differentiation. Such research will inform the development of efficacious regenerative therapies and improved clinical outcomes.

## 1. Introduction

The extracellular matrix (ECM) is a non-cellular three-dimensional network of biomolecules and minerals that provides biochemical and physical support to the cells that produce it. The ECM of different tissues varies enormously but can generally be considered as a mixture of fibrous components, including collagenous and elastic fibres, plus a gel-like ground substance, containing proteoglycans, glycoproteins, enzymes, ions, and other non-fibrous elements. The ECM is able to transmit biochemical and physical cues to cells, affecting cellular morphology and phenotype. Changes to cellular activity/function result in linked changes to the ECM, meaning that matrix cues are important drivers of tissue development, homeostasis, ageing, and disease [1,2,3,4,5].

Matrix cues are received by cells via a range of biochemical and physical mechanisms, both directly and indirectly. Biochemical cues, for example, are sensed via cell-surface receptors such as integrins and N-cadherins [6]. Physical cues, such as matrix stiffness, are transmitted most notably through the intracellular actin cytoskeleton. Stiffness, or Young’s modulus, is determined by a range of factors including the relative abundance of matrix proteins, their orientation, the degree of fibre crosslinking, and the mechanical properties of individual fibres [7]. Structural and topographical cues, including fibre size, texture, regional alignment, and orientation are additionally important factors that influence cell morphology, phenotype, and matrix synthesis and degradation. A variety of cues are also sensed by cells indirectly, through changes in nutrient transport regulation, tissue diffusion profile, and variations in hydrostatic pressure or mechanical loading. Such factors intrinsically link to other microenvironmental cues, including tissue pH, hydration, and oxygen concentration.

Understanding the impact of these various cues is of vital importance both for understanding tissue homeostasis and disease processes, and in regenerative medicine, where matrix cues may be exploited in cellular therapies to help enhance clinical outcomes. This review will therefore focus on the importance of changes to the intervertebral disc (IVD) ECM during development, homeostasis, ageing, and disease and will outline current attempts to integrate biochemical and physical matrix cues into cell-based experimental systems and regenerative therapies.

## 2. IVD Physiology, Structure, and Function

### 2.1. The IVD

The human spine contains 23 IVDs which separate the vertebral bones and whose primary functions are to absorb biomechanical forces and permit a range of motions in three dimensions [8]. The IVDs vary in size depending on their position within the spine, but the largest of the lumbar discs, found in the lower back, are approximately 4 cm in diameter and 7–10 mm thick. The IVDs retain a similar composition between disc levels [9], are generally axially symmetrical, and collectively contribute one third of total spinal height [10]. They are hydrated fibrocartilaginous structures consisting of two main regions, each contributing approximately 50% of tissue volume: first, a central gelatinous, proteoglycan-rich nucleus pulposus (NP) and second, an outer fibrous ring-like annulus fibrosus (AF). Each IVD is enclosed by two semi-rigid cartilaginous endplates (CEPs) that connect via the bony endplates (BEPs) to the vertebrae. The CEPs are thin (<1 mm) and difficult to isolate [11], so are frequently excluded from discussions surrounding the IVD; however, the CEPs are essential for nutrient transport and are believed to play a key role in maintaining IVD health [12,13,14,15]. Overall, the complex organisation of the IVD makes the organ a viscoelastic, non-linear, anisotropic structure with compressive, tensile, and shear strength in the axial and radial directions—properties highly suited to the IVDs’ primary structural function [8,16].

#### 2.1.1. The NP

The central NP is an amorphous gelatinous tissue composed primarily of water (80% wet weight) interspersed with a loose matrix of collagen type II fibrils and aggregating proteoglycans, particularly aggrecan. Other collagens (types VI, IX, and XI), proteoglycans (biglycan, versican, decorin, lumican, fibromodulin, and perlecan), laminins, fibronectin, and elastin are also prevalent [17]. The NP’s main function is to distribute compressive strain. This is principally enabled by the large quantity of proteoglycans relative to collagens (the GAG/hydroxyproline mass ratio is 27:1) [18] and a correspondingly high level of hydration. The healthy young and adult NP has a Young’s modulus under unconfined compression of 0.3–5 kPa [8,16,19,20,21,22,23,24], is viscoelastic, and has been described as a ‘tethered fluid’ [25,26]. NP tissue is maintained by a sparse population of NP cells (~4 × 10^3^ cells/mm^3^), which display a rounded, chondrocyte-like morphology [27,28] and express a range of phenotypic and functional markers, including Shh, Brachyury, KRT18/19, CA12, CD24, GLUT1, and stabilized expression of HIF1α protein [29].

#### 2.1.2. The AF

The surrounding AF tissue, in contrast, is a highly organised structure comprising 25–30 discrete concentric rings, called lamellae, which are composed principally of fibrillar bundles of collagen type I, collagen type III, and elastin [30]. The AF’s main function is to distribute tensile and shear forces whilst constraining NP swelling [31]. The thickness of the lamellae increases with distance away from the NP, ranging between 0.05 mm in the inner AF (IAF) to 0.5 mm in the outer AF (OAF) [32], whilst lamellae are also organised in an angle-ply formation, with adjacent rings orientated obliquely at 30–60° to the spine. This angle alternates between adjacent rings [33,34]. Fibre angle is elevated in the IAF compared to the OAF [35], and these structural features combined reflect the variations in mechanical loading experienced across different AF regions. Throughout the tissue, lamellae are furthermore connected by trans-lamellar cross bridges consisting of proteoglycans, most significantly aggrecan and versican, plus collagen type VI [36]. The impact these have on AF integrity and cellular behaviour has not been comprehensively studied (Table 1) [37,38], although micro-computed tomography (µCT) and other advanced imaging techniques are increasingly being applied to better understand the AF microstructure [33,39,40,41]. Overall, the complex structure of the AF confers it with excellent shear and tensile properties, with tensile Young’s modulus in the order of ~100 kPa.

The AF is maintained and populated by AF cells (~9 × 10^3^ cells/mm^3^), which display an elongated fibroblast-like morphology and express the phenotypic markers COL1A2, COL5A1, COL12A1, CD146, SFRP2, LAM1, THY1, and MKX [48,49]. The consensus regarding healthy adult AF cell phenotype remains fragile at present, although a recent study identified 1161 genes showing higher expression in AF than in NP cells [50]. Notably, there is no clear interface between NP and AF tissues; however, there is a discernible transition between IAF and OAF tissue types. Compared to OAF tissue, the IAF is richer in aggrecan, whilst collagen type I and type II fibrils are present in more equal quantities [34,51]. IAF tissue is also comparatively enriched in COL3A1, COL5A1, COL11A2, and proteins relating to matrix synthesis (PCOLCE) and remodelling (MXRA5), as well as those relating to WNT and BMP inhibition [9]. In contrast, the OAF is comparatively enriched in COL6A1/2/3, COL12A1, COL14A1, basement membrane and anchoring proteins, plus ligamentous, tendinous, and cartilaginous components, including tenomodulin and thrombospondins [9]. The presence of these particular ECM components likely reflects the OAF’s proximity and integration with the CEPs, BEPs, and ligamentous structures around the spine [14,15].

#### 2.1.3. The CEPs

The CEPs are semi-rigid layers of hyaline cartilage, approximately 0.6 mm in thickness that perform a range of functions. They have a 60% water content and an ECM rich in aggrecan and collagen type II at a ratio of 2:1 [52]. They therefore deform during mechanical loading, dissipating stress across the IVD [8], although their biochemical and physical properties are regionally dependent [53]. The CEPs also reduce the rate of water expulsion from the NP during compression, making them important regulators of diffusion; nutrients, oxygen, metabolic by-products, and other small molecules are known to diffuse through the CEPs bidirectionally [54]. The CEPs are penetrated by a thin capillary network [55] and the diffusion profile of the CEPs during mechanical loading, age-related calcification, and IVD degeneration are active areas of study, both in basic research and in tissue engineering [14,27,54,56,57,58,59,60], because of the important role the CEPs play in IVD nutritional balance. The CEPs are populated by chondrocytes (1.5 × 10^4^ cells/mm^3^) [27,28] and whilst knowledge concerning phenotype is limited, COL10 has been identified as a potential CEP cell marker in adult IVDs [61]. CEP cells have also been shown to express high levels of the NP markers Brachyury and KRT19, plus the AF cell marker MKX [62]. Given the tissue’s similarities with both NP and AF tissues, the CEPs and role of CEP-related matrix cues deserve greater inclusion in future IVD discussions and regenerative approaches; CEP matrix cues and other instructive factors are almost certainly linked to NP and AF cell behaviour and phenotype, plus overall IVD development, homeostasis, ageing, and degeneration.

## 3. Changes to the ECM during IVD Development and Degeneration

### 3.1. IVD Development and Maturation

The NP develops from the precursor foetal notochord, a soft rod-like structure composed of clusters of vacuolated notochordal cells and an ECM rich in collagens (types II, VIII, XI, XV, and XXVII), glycoproteins (laminins, fibronectin, and fibrillins), and chondroitin sulphate (CS)-rich proteoglycans, such as aggrecan. Many of these have been found to vary regionally, indicating their diverse functional roles [63]. The notochord supports musculoskeletal development and provides a series of dynamic biochemical and physical matrix cues crucial for directing IVD development [64,65,66,67]. Over time, identifiable notochordal cells are lost from the NP, disappearing almost entirely by adolescence and skeletal maturity. The mechanisms driving the loss of notochordal cells in humans remain unclear and whether the notochordal cells differentiate into, or are replaced by, NP cells continues to be a source of major contention. Recent progress in identifying phenotypic markers for notochordal [68] and NP [29] cells has revealed that NP cells are derived directly from the notochordal cell population [69]. However, subpopulations of NP [70,71] and AF [72,73] cells with potentially differing ontogenies have been identified in adult IVDs, including those of non-notochordal origin. It is therefore speculated that a small notochordal cell population persists into adulthood, producing ECM that encourages the development of a healthy NP cell phenotype and guards against the onset of early IVD degeneration [74,75,76,77].

At all stages of NP development, cell–matrix interactions are important; however, notochordal ECM is especially rich in laminin isoforms. Laminins generally act as structural proteins in both basement membrane and the notochordal sheath [66,78], but are also known to encourage NP cell clustering behaviour in developing NP tissue [7]. Notochordal and young NP cells strongly express specific laminin isoforms (LM-511, LM-521, and LM-332) and receptors (integrins α6, β1, β4, and CD239) [78,79,80], whilst laminin–cell interactions are believed to encourage cell–cell interaction, healthy cell morphology, and the proteoglycan production required for notochordal and IVD development [78]. Developmental changes within the notochord and early NP have additionally been associated with the upregulation of molecular functions linked to laminin and BMP receptor binding, strengthening the view that laminins are particularly important biochemical matrix cues relevant to developmental research and regenerative therapies.

Notochordal ECM has a lower Young’s modulus (<1 kPa) than adult NP, another factor known to encourage notochordal cell clustering behaviour and NP tissue development [7,66,80,81]. From the third to tenth weeks of embryonic development, the notochord stiffens and eventually segments, influenced by a variety of biomolecules including the intracellular transcription factors Brachyury, SOX5/6, and PAX1, plus secreted morphogens and signalling molecules BMP 2/7 [82], Shh, and Noggin [83]. The 23 NP regions eventually result from this variety of interconnected cues and further research is required to understand the full relevance of the spatial and temporal changes inherent to such cues as they drive development.

In contrast, the adult AF and CEP regions are of mesenchymal origin, developing from sclerotomal tissue surrounding the foetal notochord. From around the fifth week post-conception, this sclerotomal tissue condenses and expands, encompassing the notochord, whereupon elastin, fibrillin, and perlecan influence the formation of collagenous attachments between nascent AF lamellae and the cartilage and vertebral bodies [83,84]. Knowledge of the impact of cell–cell and cell–matrix interactions on AF development lags significantly behind that of NP and the notochord; however, it is suspected that the organisation of the AF is specified by physical cues present during early development. For example, the number of lamellae is fixed during early foetal development, with fibres subsequently thickening and increasing in volume according to the physical and geometrical stresses induced by the stiffening notochord and surrounding ECM [85]. It is furthermore proposed that early fibre organisation informs the development of intracellular actin stress fibres within developing AF cells [86], which in turn may guide the production of aligned ECM and help redistribute mechanical loading as the IVD develops. Intriguingly, the orientation and integration of AF fibres into the BEPs, CEPs, and surrounding tissues appears to be driven by physical constraints caused by the emergence of the endplates and vertebral bodies [34,85,87], implying that physical matrix cues are central to AF development.

### 3.2. IVD Degeneration

Degeneration is a broad term that encompasses the myriad changes taking place within the IVD, which ultimately result in a loss of structural integrity, pain, and an overall reduction in quality of life. Many of the changes that occur in the IVD due to age and degeneration are indistinguishable from one another, and as a result, the root causes of degeneration remain only partially understood. It is widely accepted, however, that a combination of mechanical loading, genetic factors, and reduced cellular activity are all responsible for the changes in matrix production that lead to the loss of tissue homeostasis and increased matrix degradation typically observed with degeneration [75,88,89,90,91,92,93,94,95].

The healthy IVD is avascular, with the possible exception of the extreme OAF, and so cells must maintain tissue health by balancing matrix anabolism and catabolism within each disc region. A variety of anabolic and anti-catabolic factors are therefore important for NP and AF health, including those from the TGFβ superfamily, matrix metalloproteinases (MMPs), aggrecanases/a disintegrin metalloproteinase with thrombospondin motifs (ADAMTS), and tissue inhibitors of metalloproteinases (TIMPs), along with a number of genes relating to structural proteins correlated with IVD degeneration. Damage-associated molecular patterns (DAMPs), including hyaluronic acid and fibronectin fragments, are also believed to be primary contributing factors to the inflammation and pain experienced by most sufferers of IVD degeneration [96,97].

**Figure 1:**
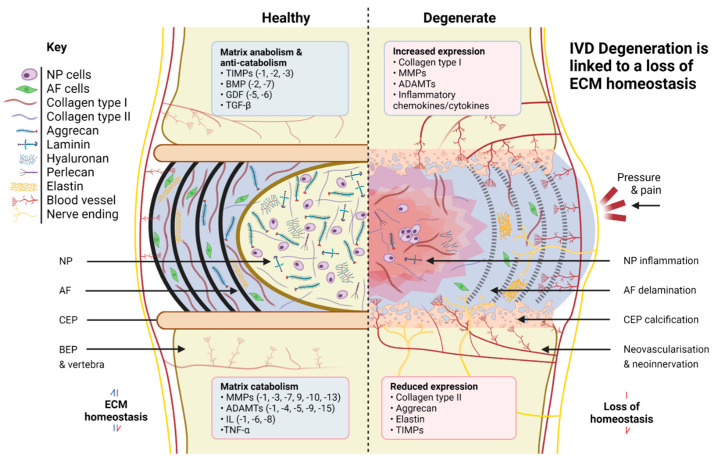
Comparison of healthy and degenerate IVD ECM. Created with Biorender.com.

Degeneration typically begins within the NP region, where an upregulation of inflammatory cytokine production leads to the downregulation of matrix synthesis, especially aggrecan and collagen type II. This shift coincides with an increase in matrix degradation via upregulation of matrix degrading enzymes (MMPs/ADAMTS), which results in proteoglycan fragmentation [98] and a corresponding loss in the ability of the tissue to retain water [99,100]. The linked loss in hydrostatic pressure leads to an overall reduction in disc height, which impairs the IVD’s overall mechanical integrity and encourages further degeneration, the start of a vicious cycle [101]. The NP also experiences a shift in the production of collagens, from predominantly collagen type II to the more fibrous type I, resulting in increased ECM stiffness, a key driver of NP degeneration [102,103] (Figure 1).

There is a growing body of work documenting the negative impact of increased stiffness on both NP and notochordal cell phenotype [7,66,104,105]. Interestingly, degeneration of the NP coincides with the loss of identifiable notochordal cells, a phenomenon observed spontaneously in many species, including humans and chondrodystrophic dogs, but not in pigs, rabbits, or non-chondrodystrophic dogs. Species which retain their notochordal populations do not generally suffer from significant IVD degeneration [103], reinforcing the view that notochordal cells are a promising target for regenerative therapies. Work is ongoing to determine the roles of master notochordal cell regulators and to identify how best to use these for the detection of early degeneration and to retard disease progression [105,106,107,108,109] using novel regenerative therapies.

Overall, such observations suggest physical matrix cues are important contributors to the initiation or progression of degeneration. Such a view is reinforced by changes occurring in the AF during degeneration, where the impacts appear mainly mechanical. For example, additional mechanical stress due to NP degeneration can lead to AF microfissuring and a widening of the interlamellar spaces, meaning lamellae lose their organisation and orientation due to load redistribution [102]. Stiffening of AF tissue is another contributing factor to this structural deterioration; however, it is the loss of aggrecan which particularly impacts AF health, since aggrecan is anti-angiogenic and its absence leads to neovascularisation [110]. AF vascularisation results in increased oxygen concentration and disrupted signalling pathways, and can be responsible for driving AF and NP cell senescence [111], further impairing tissue homeostasis and overall mechanical integrity. In the most serious instances of AF degeneration, herniation of the NP through the AF may even occur, permanently fissuring the already weakened AF structure [112,113].

Degenerative changes also take place in the CEPs and adjacent vertebral structures, although the significance of many of these remains unclear. Degeneration-associated CEP calcification is known to be important [13,14,56,60], as a loss of CEP permeability results in reduced nutrient transport and altered CEP mechanical profile [114], plus knock-on effects for NP and AF cells. These include cellular quiescence [115,116], deregulation of matrix anabolism and catabolism [117], and a reduction in the release of exosomes, recently discovered to inhibit IVD degeneration and NP cell apoptosis [118,119]. Nerve ingrowth and neovascularisation through the CEPs into the AF are additional disease pathologies directly linked to pain [120], highlighting the links between the condition of the CEPs and other tissue regions, as well as the importance of understanding the range of matrix cues spanning all three regions as these change over time.

Transcriptomic and proteomic analyses have begun to reveal many of the IVD-wide and more subtle spatiotemporal changes in genes and proteins that may result in downstream regulation of matrix anabolism and catabolism or influence cell phenotype and matrix production, both in non-humans [73,121,122,123,124,125,126,127] and in humans (Table 2). Collectively, they have additionally identified age-related compositional differences and regionally specific variations in matrix proteins [128], indicating that some ageing and degenerative mechanisms act only locally within NP and AF tissues, as opposed to at the organ-wide level. Such novel approaches open up vast new avenues of study and will transform our understanding of the importance of different transcription factors influencing matrix cues during degeneration. In turn, this will allow the accelerated development of degenerative models and novel therapies incorporating a variety of spatiotemporally defined matrix cues.

## 4. Incorporation of Matrix Cues into Experimental Models Using Biomaterials

### 4.1. The Need for Regenerative Therapies

Back pain is one of the leading causes of disability worldwide. In the UK alone, it has an estimated socio-economic cost of £12 billion [144], whilst in the USA the total impact may be as high as $100 billion [145]. It is predicted that 85% of individuals from Western societies will experience back pain at some point in their lives [146], and an estimated 632 million people are suffering from the condition at any one time [147]. However, treatments focus principally on pain management and non-invasive interventions such as physiotherapy. Intradiscal injections and the removal of severely degenerate discs are also common [148], whilst grafting and novel prosthetic implants are increasingly yielding superior clinical outcomes [149]. However, such innovations, whilst welcome, ultimately fail to address IVD degeneration and are reported in many instances to accelerate the breakdown of adjacent IVDs post-treatment [150,151,152,153]. The long-term prognosis for surgical procedures is thus unsatisfactory [154,155]. Efforts to physically repair degenerate IVDs, particularly through the use of AF sealants and fibrous composites, are progressing [156,157,158], but it is novel biological approaches that hold the most promise, including platelet-rich plasma (PRP) therapy [159,160,161,162,163], gene therapy, and cell-based therapies. Gene therapy, a broad class of treatments involving the genetic modification of cells for therapeutic effect, is proposed as the key to long-term inhibition of IVD degeneration [164,165]. Recent advances involving gene silencing via RNAi, gene editing using CRISPR, and the delivery of non-viral vectors to cells have hinted at the feasibility of targeting the inflammatory receptors of degenerate IVD cells to inhibit degeneration by limiting matrix catabolism [166]. However, the field is still in its early phases, and many basic questions remain regarding clinical efficacy, cost-effectiveness, and long-term ethical implications.

### 4.2. Cell-Based Regenerative Approaches

Cell therapy, the implanting of cells into diseased tissue, offers another exciting regenerative strategy with real potential for IVD regeneration. Autologous and allogenic notochordal [167], NP [168], AF [169], chondrocyte [170], and pluripotent/multipotent stem cells [171,172,173,174,175,176,177,178] have all been trialled in cell-based therapies [17], with experimental objectives including to limit production of inflammatory cytokines, encourage matrix anabolism, and repopulate or stimulate native IVD cells [179]. All these cell types appear to have properties suitable for IVD regeneration. Whilst in vitro evidence is strong and there is compelling evidence from in vivo studies, current clinical trial data do not yet provide high quality evidence of efficacy [180], highlighting the need to promote appropriate cell function or, in the case of stem cells, lineage commitment. Short-acting growth factors are occasionally employed to stimulate cell function [181], but their high cost and the risk of off-target effects means that most cell therapies do not use growth factors, and cells are instead injected in isolation. Cells therefore lack many of the supporting biochemical and physical cues known to drive healthy phenotypes or discogenic differentiation and may instead receive abnormal cues linked to degeneration, limiting their regenerative potential. Cells sourced from or delivered to degenerate tissue show a strong tendency to de-differentiate or produce degenerative phenotypes, either due to a lack of cell function or because of cell death in the harsh microenvironment of the degenerate IVD [182,183,184]. This highlights the need to identify the most appropriate matrix cues to deliver to cells, in order to better control and direct their behaviour and health, along with ECM restoration.

If cell-based therapies are to improve, understanding how best to deliver these cues and minimise the impact of the degenerate IVD microenvironment is of vital importance. Comparatively little work has focused on how different cell types respond to the matrix cues presented by the degenerate IVD environment. These cues are substantially different from those in healthy IVD tissue, which are themselves different from those of the developmental IVD and foetal notochord, and whether the incorporation of ‘healthy’ matrix cues can be used to promote a healthy phenotype or drive more appropriate stem cell differentiation and matrix formation is an open question. There is therefore a real need to provide the cells used in cell therapies with matrix cues through the use of biomaterials in experimental models and in clinical regenerative therapies. The designing of systems that can deliver regionally specific instructive cues to cells is one of the priorities facing IVD regenerative research moving forward. If achieved, models can be developed that mimic IVD development, health, and degeneration, allowing the study of the role of specific matrix cues and the inclusion of cues into regenerative therapies, with the aim of restoring appropriately functioning ECM and reducing or eliminating pain.

### 4.3. Development of Biomimetic Systems for the Delivery of Matrix Cues

Research is ongoing to develop biomaterials and systems that can deliver biochemical and physical cues to cells and yield experimental insights regarding the roles of specific instructive cues within the IVD. The most notable body of work in this regard is the development of a library of laminin-mimetic peptide hydrogels for directing the NP cell phenotype in 3D. At least six integrin- and syndecan-binding peptide sequences have been developed which successfully drive healthier NP cell phenotypes [132]. The use of such systems provides the clearest indication yet that matrix cues can be used to directly modulate cell behaviours [185] and potentially mimic a combination of cues present in development, health, and degeneration. Peptide-conjugated alginate hydrogels have similarly been exploited, with cell-adhesive and syndecan-binding domains encouraging the production of NP-specific phenotypes within alginate cultures, in some instances regardless of presented cell-adhesive domains [186]. Such systems can in the future be used to determine differences in transduction experienced by young/healthy and degenerate cell types and the importance of matrix cues and receptor interactions [186].

A number of separate biomaterials systems incorporating collagen-based instructive cues are also in development and have been shown to modulate cell phenotype and differentiation. For NP, collagen type II crosslinked with genipin has been used to promote the differentiation of adipose-derived stem cells (ADSCs) into NP-like cells via the Shh pathway [187], whilst genipin has been employed to stabilise a collagen type II and chondroitin sulphate gel capable of encouraging healthy NP-like expression in ADSCs and the partial restoration of NP [188]. A denatured form of collagen, gelatine, was also combined with hyaluronic acid and methacrylate in a photo-crosslinkable hydrogel to achieve NP-like differentiation of ADSCs and the reversal of degeneration in vivo [189], whilst, in one instance, type II collagen was combined with both hyaluronic acid and chondroitin sulphate in a rabbit degeneration model to encourage disc cell repopulation and matrix production [190]. For AF, scaffold materials containing collagen type I are common, increasingly within stiffness-tuneable materials [191,192,193] and with the addition of cells and growth factors [194]. An MSC-laden collagen type I gel was recently applied to reverse AF degeneration, albeit in sheep [195], whilst AF-derived stem cells have been trialled in a collagen type I-containing decellularised ECM (dECM) [196]. Many relevant collagen-based systems exist that have not yet been applied to IVD bioengineering; for example, combined collagen type I and type II blended hydrogels [197] with chondroitin sulphate [198] for MSC differentiation and articular cartilage repair, hinting at the use of combinations of matrix molecules simultaneously in NP, AF, and CEP research in future. This growing body of work is important, as it indicates collagen-based biomaterial culture systems can be designed to specifically deliver type I and type II-based instructive cues for the directing of cell phenotype and healthy matrix production, potentially in combination with glycosaminoglycans (GAGs) and other collagens [199,200].

Aggrecan, hyaluronic acid, and other GAG-based hydrogel systems and their biomimetic equivalents are needed moving forward. There is significant research in this regard applied to cartilage engineering [201,202,203,204,205,206,207,208,209,210], including systems that can be localised within ECM tissue through the incorporation of HA-specific binding peptides [201,205,206] and via steric interactions with collagen type VI and perlecan [211]. Some of these systems have been applied to the IVD [212], most notably in the case of a cytocompatible large aggrecan mimic, which has been chemically, structurally, and mechanically characterised and injected ex vivo into bovine NP tissue [211]. Proteoglycan-like systems principally mimic or incorporate heparan sulphate [213] and the chondroitin sulphate chains of aggrecan in order to confer a hydrating function [214,215]. However, they have also been used to aid differentiation [216] and deliver growth factors to mesenchymal stem cells (MSCs) [217], plus encourage collagen and GAG production in MSCs for NP regeneration [218]. Collectively, these studies demonstrate new opportunities for macromolecular matrix engineering that have potential to alter the degenerate IVD microenvironment and augment cellular therapies with growth factors, tissue engineered scaffolds, and the delivery of instructive matrix cues [211] (Table 3).

dECM-based alternatives have also advanced in recent years, although the bulk of research has focused on cartilage and bone tissue engineering applications, not IVD. Tissue-specific dECM is regarded as a promising alternative to other biomimetic matrix systems as they may enable a more accurate reflection of the native tissue environment [219,220], delivering collagens, proteoglycans, laminins, and other important matrix components [221]. In the case of IVD, dECM biomaterials have been used to provide scaffold materials and growth factors to AF-derived stem cells [196] and MSCs co-cultured with degenerate NP cells [222]. dECM biomaterials are increasingly being developed as scaffold coatings, within hydrogels, and for bioinks in 3D bioprinting [223,224,225,226,227,228].

**Table 3:** Matrix molecules incorporated into biomimetic systems for IVD.

Matrix Cues(s)	Study Outcome	References
Type I collagen	Injectable collagen gel upregulates aggrecan and collagen type I production for in vitro AF repair.	[192]
	Alginate-collagen porous scaffolds supported MSC proliferation and collagen type I production.	[193]
	Injectable high-density collagen gel partially repaired AF defect and remodelled by host fibroblasts into a fibrous cap.	[191]
	Injectable high-density collagen gel seeded with MSCs resulted in increased disc height, reduced Pfirrmann grade, and increased NP area.	[195]
	Injectable TGFβ1-supplemented collagen hydrogel resulted in production of collagen type I, CD146, MKX, and SM22α.	[194]
Type I collagen and HA	Injectable collagen-HA hydrogel enabled growth factor delivery and supported growth and chondrogenic differentiation potential of MSCs and nasal chondrocytes.	[229]
Type II collagen	Injectable, crosslinked collagen hydrogel enriched with HA preserved NP cell morphology.	[230]
	Collagen microspheres aided pre-differentiation of ADSCs in degenerate IVD-like conditions.	[189]
	Collagen scaffold activated the Shh pathway in ADSCs, promoting NP-like differentiation.	[187]
Type II collagen and HA	Microgels influenced ADSCs to express high levels of collagen type II, aggrecan, and SOX9, and low levels of collagen type I.	[231]
	Type I or II with HA hydrogels identified the role of SOCS in combating pro-inflammatory cytokine effects in degenerate NP.	[232]
Type II collagen and CS	CS incorporation resulted in increased production of NP-like ECM, including sulphated GAGs.	[233]
Type II collagen and HA and CS	Cell-seeded scaffolds supported NP cell viability and resulted in maintenance of disc height.	[190]
Laminin	Injectable laminin-111 functionalized poly(ethylene glycol) (PEG) hydrogel resulted in significantly higher cell retention within NP.	[234]
Laminin mimic	Laminin-mimetic peptides resulted in cell signalling downstream of integrin and syndecan binding, promoted cell migration, and modulated NP behaviour similarly to full-length laminins.	[185]
	Integrin- and syndecan-binding peptide-conjugated alginate hydrogel elicited NP-specific phenotype and re-expression of more juvenile-like phenotype in NP cells.	[186]
	Integrin- and syndecan-binding peptide-conjugated alginate hydrogel used to identify novel mechanosensitive targets in NP cells, including several G Protein-Coupled Receptor genes.	[132]
HA	HA-pNIPAM hydrogel induced greater disc-like differentiation of MSCs compared to pre-differentiation, including collagen type II, SOX9, KR19, and CD24.	[235]
	Eight different HA-PEG hydrogel formulations used to identify key parameters influencing IVD cell–material interactions.	[236]
	Treatment with HA-based hydrogel resulted in downregulation of NGF and BDNF, plus suppression of IL1R1 in an in vitro inflammation model of NP.	[237]
	HA and PRP hydrogel blended with batroxobin gelling agent resulted in heightened sulphated GAG production in MSCs and chondrocyte-like differentiation.	[218]
	Assessed the efficacy of HA hydrogel pain alleviation, demonstrating altered glycosylation plus modulation of inflammatory and regulatory signalling pathways.	[238]
	HA oligosaccharides shown to stimulate MMPs, ADAMTs, and anabolic matrix repair genes.	[239]
	Gelatine-HA hydrogel promoted NP-like differentiation of ADSCs.	[188]
HA and GAG mimic	Used HA-PEG hydrogels to demonstrate the chondro-inductive potential of pentosan polysulphate, a sulphated semi-synthetic polysaccharide.	[240]
GAG mimic	Cytocompatible biomimetic aggrecan analogue comprising a polymeric core conjugated to CS ‘bristles’ was shown to have comparable osmotic pressure to natural aggrecan and increase intradiscal pressure upon injection.	[212]
	Pentosan polysulphate embedded within a gelatine-fibrin scaffold with MSCs resulted in restored disc height, morphology, and NP proteoglycan content.	[241]
	Cytocompatible, large biomimetic aggrecan analogue comprising a polymeric core conjugated to CS was chemically, structurally, and mechanically characterised. Injection into ex vivo bovine NP showed localisation in the pericellular matrix.	[211]
	Co-polymerised naAMPS and KSPA GAG mimic provided intrinsic swelling pressure and restoration of stiffness within ex vivo NP.	[214]
Elastin	Bioprinted silk fibronin hydrogel combined with elastin shown to support ADSC culture and enable creation of scaffolds with structural and mechanical properties similar to AF.	[242]
Matrilin-3	Gelatine microparticles loaded with TGFβ3 and matrilin-3 promoted chondrogenic differentiation of ADSC spheroids while preventing hypertrophy and terminal differentiation of cells.	[243]
Decellularised AF ECM	Decellularised ECM and chitosan hydrogels increased production of collagen types I and II, and aggrecan, in AF-derived cells.	[196]

The development of biomaterials capable of delivering variations of combined cues to guide cell behaviour is a research bottleneck, and there are unfortunately no systems that can yet deliver a comprehensive range of the many physiologically relevant biochemical cues so far identified, including those that are laminin-, collagen-, aggrecan-, and GAG-based. Nor are there many that combine the use of these matrix molecules with stiffness-tuneable biomaterials for mechanical cue incorporation. This has been achieved in a small number of instances for IVD, using in situ crosslinkable peptide delivery systems [244,245] and laminin-functionalised pegylated hydrogels of varying stiffnesses [185]. Such approaches are generating novel data regarding mechanotransduction, notochord morphogenesis, intracellular signalling, cell differentiation, and matrix synthesis, which are of major relevance to IVD regeneration strategies moving forward [81,132].

As the range of biomaterials systems expands, the inclusion of comparatively unexplored molecules known to have matrix regulatory functions is an important step. For example, perlecan is speculated to directly impact gene regulation and matrix stabilisation within IVD cells [246,247]; however, its role in regulating healthy and degenerate phenotypes and stem cell differentiation remains unclear [248,249]. The impact of early protein and proteoglycan deposition on cellular behaviour within hydrogels has also largely been overlooked, with recent studies showing that the interface between newly produced pericellular matrix and biodegradable/dynamic hyaluronic acid-based hydrogels modulates MSC differentiation with some correlation to hydrogel crosslinking density [250,251]. This implies the effect of matrix cues may vary over time, with short-term impact heightened and long-term impact reduced, although such aspects are not well understood. It may be that spatial variations are important, with cues normally localised in the pericellular matrix having a more profound effect than cues from the inter-territorial matrix. The importance of laminin cues may be a clear example of this since cells interact more closely with laminins than collagen type II or aggrecan. Experimental systems must be designed that can determine exactly which biomaterial cues are masked in the short- and long-term and how these cues can be exploited through spatial and temporal patterning to direct regional effects.

One of the main inhibitors of progress in this regard is the difficulty of achieving spatiotemporal control over stiffness and viscoelasticity in existing hydrogel systems, along with spatiotemporal presentation of biochemical cues. Graphene-based delivery systems have been used to provide sustained local delivery of growth factors and have additionally been incorporated within collagen hydrogels for cartilage engineering and MSC differentiation [252,253]. Work could focus in the short-term on the development of small molecule delivery systems such as graphene-based and BMP2/7-, GDF5/6-, and TGFβ-laden hydrogels [253] to help understand cell modulation and which cues are masked. In the longer-term, once comprehensive matrix instructive systems exist, work would be needed to replace growth factors entirely using a combined selection of more stable matrix cues.

### 4.4. Spatially Controlled Patterning of Matrix Cues Using Biofabrication Strategies

Advanced manufacturing techniques are increasingly being applied to model complex tissues and pattern matrix molecules with the aim of achieving spatiotemporal control over matrix cues, including electrospinning and 3D bioprinting. Electrospinning uses high-voltage electric fields to project polymer fibre melts and solutions onto charged surfaces at varying length scales and can produce highly aligned multi-layer fibre scaffolds [254]. The technique is therefore a popular tool for replicating AF fibre organisation, plus scaffolds for AF tissue engineering, both acellularly and in combination with AF and stem cells [255,256,257,258,259]. The technology has also been applied to model the AF-CEP interface [260], an intriguing approach that exploits electrospinning’s capabilities particularly well. Notable attempts have been made to investigate and maintain the phenotype of AF and stem cells in electrospun multi-lamellated IAF and OAF constructs by varying fibre diameter, orientation, and other physical cues [261,262,263], revealing much about the possible structure–function relationship of AF tissue and its component cells. For instance, AF-derived stem cells seeded on aligned polyurethane (PU) scaffolds have been shown to become elongated and better aligned, exhibiting heightened production of collagen type I and aggrecan compared to cells cultured on non-aligned scaffold [264]. AF-derived stem cells have additionally been shown to respond to fibre size, with AF-like morphology and phenotype promoted by the presence of larger fibres [265,266]. Electrospun scaffolds have also helped shed light on the stiffness- and topographical-dependency of Yes-associated protein (YAP) in AF-derived stem cells, hinting at electrospinning’s ability to modulate stem cell behaviour using structural and physical matrix cues [267,268].

It is notable how few attempts there have been to use electrospun systems incorporating biochemical or physical matrix cues within an IVD context [268]. Electrospun fibres containing dECM have been shown to be beneficial for the creation of scaffolds for cartilage tissue engineering; however, such outcomes have yet to be comprehensively demonstrated for IVD [269]. One of the only clear examples of biochemical cue inclusion using this method was achieved by blending TGFβ into a polymer scaffold solution before electrospinning. Seeded AF cells subsequently produced greater amounts of GAGs and collagens than when scaffolds lacking TGFβ were used [270]. More of this is needed moving forward, preferably with core matrix components as opposed to growth factors, although this is a technical challenge. It must be considered, however, that whilst electrospinning is a valuable tool, the technology is ultimately inappropriate for designing whole tissue engineered IVD models in a research or clinical setting. The creation of electrospun IVD models traditionally relies on the manual rolling of the AF and injection of an NP-like hydrogel, a labour-intensive process which introduces discontinuities between regions [271,272,273], whilst the stiffness of electrospun materials has generally been an order of magnitude higher (~MPa) than native ECM (~kPa). Any mechanical cues transmitted to cells are therefore not representative of those present in the native environment, even if the models are structurally useful. Nevertheless, several electrospun constructs have been implanted into small and large animal models [192,274,275], and electrospinning is likely to remain a core technique, both for basic IVD research and for the creation of total disc replacement devices and other tissue-engineered approaches.

An alternative scalable approach used to model NP, AF, and whole IVDs is 3D bioprinting. Bioprinting is an umbrella term for a powerful set of 21st century techniques including vat photopolymerisation, material jetting, and extrusion-based bioprinting, which allow the precise spatial delivery of cells, matrix components, growth factors, and biomaterials [276,277,278,279]. This establishes bioprinting as an invaluable tool for the creation of multi-material ex vivo culture systems for basic research. The printing of low-viscosity materials such as hydrogels, whilst beneficial for cell culture, remains a challenge, and the shape fidelity of printed constructs is often compromised. To ensure fidelity and to main high cell viability, strategies such as Freeform Reversible Embedding of Suspended Hydrogels (FRESH) and Suspended Layer Additive Manufacturing (SLAM) have been developed to restrict fluid flow and improve printability [280,281,282,283,284]. These technologies could in theory allow the patterning of matrix cues in specific locations and concentrations within bioprintable hydrogel systems. IVD cells have been bioprinted to model NP and AF tissue using shear-thinning hydrogels; however, the development of biochemically and physically relevant printable biomaterials for IVD is a major bottleneck [285], particularly when modelling the stiffer AF region. To address this, hydrogels, including gelatine- [286] and gellan gum-based [287], have been co-printed with PCL scaffolds, increasing the stiffness of the bioprinted constructs. In one instance, CTGF and TGFβ were included [286], demonstrating that growth factors could be delivered in a spatially controlled manner and used to induce bone marrow stromal cell differentiation towards NP and AF cell phenotypes in different disc regions. Several attempts have also been made to bioprint hydrogel scaffolds that mimic the AF’s lamellar structure and the changing biochemical and physical properties across the OAF, IAF, and NP interfaces [269,288,289,290,291,292,293]. It is therefore possible to foresee the creation of whole IVD models for basic and clinical research, with IVD constructs being designed with the aid of patient MRI data [294,295].

It seems likely that bioprinting will be applied in combination with other advanced manufacturing technologies such as melt electrospinning direct writing (MEDW), the layer-by-layer assembly of melted fibres. This combined approach, sometimes termed hybrid bioprinting [296], has the potential to deliver matrix-reinforced cell-seeded hydrogels with tailored mechanical properties [297,298]. Whilst much of this research is in its early stages, the design and fabrication of hybrid MEDW-hydrogel systems for cartilage engineering is of particular relevance to IVD researchers, as there are several examples of chondrocyte- and ADSC-based systems [299,300,301,302] that could serve as inspiration for IVD models incorporating matrix cues. Since the importance of many matrix cues for IVD health and discogenic differentiation has been established (Table 1), the application of these cues within multiphasic biofabricated IVD and stem-cell-based systems seems a logical next step. The results of these experiments could pave the way for the creation of a series of developmental, healthy, and degenerate IVD models where the controlled use of relevant matrix cues leads to the design of regenerative therapies with better clinical outcomes.

## 5. Conclusions

The developing, healthy, and degenerate IVDs are primarily composed of ECM. Understanding the influence of biochemical and physical matrix cues on cellular activity and matrix production is therefore vital for the development of better clinical therapies targeting back pain. Lately, progress has been made in the design of experimental systems capable of delivering key matrix cues. The development of laminin- and proteoglycan-like mimics is a particularly encouraging step, although the development of biomaterials able to deliver spatially and temporally controlled cues remains a major challenge moving forward. Tissue-engineered systems, created using electrospinning and 3D bioprinting, are increasingly being used to create structurally relevant NP, AF, and CEP models incorporating a range of disc- and stem-cell types, and this enables the investigation of regionally specific matrix cues in vitro and in vivo within whole IVD systems. In the future, efforts should focus on the development of bioprintable materials capable of delivering physiologically relevant biochemical and physical matrix cues in combination with other advanced manufacturing techniques such as MEDW. If this is achieved, the effect of individual matrix cues can be better understood and employed in the design of efficacious regenerative therapies.

## Figures and Tables

**Table 1:** Composition and biological function of key matrix components within healthy adult IVDs [8,28,42,43,44,45,46,47].

Tissue	Matrix Component	Relative Composition (Dry Weight)	Structural/Biological Effects
NP	Collagen (esp. type II)	5–20%	Confine hydrating proteoglycans within NP matrix. Modulation of cellular metabolism and extracellular signalling.
	Proteoglycans (esp. aggrecan, versican, biglycan)	35–65%	Generate swelling pressure; influence expression of NP cytoskeletal elements and matrix anabolism/catabolism during compression, particularly collagen fibrillogenesis.
	Non-collagenous proteins (esp. laminin)	15–60%	Play a key role in matrix organisation through interaction with integrins.
AF	Collagen (esp. type I)	50–70%	Fibre orientation and composition ratio influences biomechanics and matrix production within IAF/OAF.
	Proteoglycans (esp. aggrecan, versican, decorin)	10–20%	Generate swelling pressure; influence expression of AF cytoskeletal elements and matrix anabolism/catabolism during tension and shear; regulate matrix assembly and repair following damage.
	Non-collagenous proteins (esp. elastin)	10–40%	Support development of elastic matrix and enable collagen fibre recovery after deformation.

**Table 2:** Important matrix genes and proteins identified during IVD omics studies in humans.

Source	Method	Matrix-Related Genes/Proteins Investigated	References
Transcriptomic			
Foetal/notochord	Microarray analysis	Identified CD24, IGF1 and eight other notochord-specific markers, plus molecules involved in inhibiting vascularisation (WISP2, Noggin, and EDN2) and inflammation (IL1-RN).	[105]
Foetal/notochord	scRNA-seq	Identified eight drivers of notochordal differentiation: PAX6, GDF3, FOXD3, TDGF1, SOX5, LMX1A, LEFTY1, and LEFTY2.	[109]
Young/healthy NP	Microarray analysis	Identified marker genes including PAX1, FOXF1, HBB, CA12, and OVOS2 for NP; GDF10, CYTL1, IBSP, and FBLN1 for articular chondrocytes.	[129]
Young/healthy NP and AF	scRNA-seq	Higher expression of COL2A1, COL9A3, and COL11A1 in NP. Signature transcription factor for NP (KDM4E) and AF (FOXM1) identified.	[130]
Young/healthy NP, AF, and CEP	scRNA-seq	Identified progenitor IVD cell markers plus PDGF and TGFβ cascades important for regulating NP microenvironment.	[131]
Young/healthy and degenerate NP, IAF, and OAF	Microarray analysis	Overall, showed relative enrichment of COL5A1, SERPINA5, and MXRA5 in IAF; LAMB2, THBS1, and CTSD in OAF. Identified that clearest transition in proteomic signature is between ECM of OAF and IAF, not between IAF and NP.	[9]
Degenerate NP	RNA-seq	Used integrin- and syndecan-binding laminin-mimetic peptides to upregulate 148 genes, including NP markers (Noggin and ITGA6), and downregulate 277 genes, including known fibroblastic markers linked to matrix catabolism (CTGF).	[132]
Degenerate NP and AF	RNA-seq	High expression of genes encoding proteoglycan-rich ECM and TGF superfamily signalling pathways in NP (GPC6, INHBA, INHA); fibrous, vascular matrix and WNT/NOTCH signalling in AF (COL1/4/6, VEGFC, and JAG1/2).	[133]
Immortalised NP and AF cell lines	Microarray analysis	Identified membrane-associated genes for cellular subtypes, including CLDN11, TMEFF2, EFNA1 and NETO2 for NP; COLEC12, LPAR1, and CHIC1 for AF. Indicated that regulation of WNT signalling separates AF from NP cells.	[50]
Proteomic			
Foetal NP	LC-MS/MS	Identified 1316 proteins, 1096 of them unique, and 10 significant protein clusters including collagens, SLRPs, and matrilins. Highly expressed COL14A1 identified for the first time.	[134]
Foetal/notochord and degenerate NP	LC-MS/MS	Identified 12 degeneration-linked proteins including interleukin-11, LTA, ECM1, and matrilin-3.	[135]
Foetal/notochord, young/healthy, and degenerate NP	ESI–LC–MS/MS	Identified ten ECM regulators and ECM affiliated proteins of interest, including four (P4HA1, PLOD1/2, and SERPINH1) involved in collagen biosynthesis.	[136]
Young/healthy and degenerate AF	MS and silver-stained 2-D electrophoresis gels	Degeneration led to decreases in three proteins and increases in seven, suggesting possible degenerative biomarkers and loss of cell adhesion ability.	[137]
Young/healthy NP and AF	LC-MS/MS and iTRAQ analysis	High level of lubricin and low levels of biglycan compared to seven other cartilaginous tissue types.	[138]
Young/healthy and degenerate NP and AF	LC-MS/MS and iTRAQ analysis	Increased levels of CILP and CILP2 in NP; HTRA, COMP, and CILP in AF with degeneration.	[139]
Young/healthy IVDs	Peptide location fingering	Indicated age-related structural differences in over one hundred ECM-associated proteins including COMP, CILP, CILP2, and LRP1. Regionally specific variations in collagen type II and type V, and aggrecan across ages.	[128]
Young/healthy and degenerate IVDs	LC-MS/MS	Observed reduction in structural and other matrix proteins including COL2A1, KRT, BGN, VCAN, and DCN with degeneration.	[140]
Degenerate NP and AF	FTMS/ITMSMS and iTRAQ analysis	Fifty-four and seventy-three proteins differentially regulated in NP and AF, including integrin-mediated cell adhesion pathways.	[141]
Bone marrow-derived stem cells (BMSCs) exposed to young/healthy and degenerate IVD environment	LC-MS/MS	Altered regulation of 224 and 223 proteins following exposure to healthy or degenerate IVD microenvironments compared to baseline secretome. Following trauma, MMP and IL production observed; however, CTGF, LTBP2, and TIMP1 were also recorded, indicating attempted inhibition of matrix degradation and inducement of NP cell growth and matrix production.	[142]
Metabolomic			
DegeneraH HR MAS NMR te NP and AF	^1^H HR MAS NMR spectroscopy	Correlation between degree of degeneration and metabolites including glycine and hydroxyproline, associated with significant collagen breakdown. Reduced abundance of CS observed in highly degenerate specimens.	[143]

## Data Availability

Not applicable.

## References

[1] Carvalho M.S., Alves L., Bogalho I., Cabral J.M.S., da Silva C.L. (2021). Impact of Donor Age on the Osteogenic Supportive Capacity of Mesenchymal Stromal Cell-Derived Extracellular Matrix. Front. Cell Dev. Biol..

[2] Birch H.L., Harris J.R., Korolchuk V.I. (2018). Extracellular Matrix and Ageing. Biochemistry and Cell Biology of Ageing: Part 1 Biomedical Science.

[3] Di Loreto R., Murphy C.T. (2015). The cell biology of aging. Mol. Biol. Cell.

[4] Sun Y., Li W., Lu Z., Chen R., Ling J., Ran Q., Jilka R.L., Chen X.D. (2011). Rescuing replication and osteogenesis of aged mesenchymal stem cells by exposure to a young extracellular matrix. FASEB J..

[5] Kurtz A., Oh S.J. (2012). Age related changes of the extracellular matrix and stem cell maintenance. Prev. Med..

[6] Hwang P.Y., Jing L., Michael K.W., Richardson W.J., Chen J., Setton L.A. (2014). N-Cadherin-Mediated Signaling Regulates Cell Phenotype for Nucleus Pulposus Cells of the Intervertebral Disc. Cell. Mol. Bioeng..

[7] Gilchrist C.L., Darling E.M., Chen J., Setton L.A. (2011). Extracellular matrix ligand and stiffness modulate immature nucleus pulposus cell-cell interactions. PLoS ONE.

[8] Newell N., Little J.P., Christou A., Adams M.A., Adam C.J., Masouros S.D. (2017). Biomechanics of the human intervertebral disc: A review of testing techniques and results. J. Mech. Behav. Biomed. Mater..

[9] Tam V., Chen P., Yee A., Solis N., Klein T., Kudelko M., Sharma R., Chan W.C.W., Overall C.M., Haglund L. (2020). Dipper, a spatiotemporal proteomics atlas of human intervertebral discs for exploring ageing and degeneration dynamics. eLife.

[10] Broberg K.B. (1983). On the mechanical behaviour of intervertebral discs. Spine.

[11] Berg-Johansen B., Han M., Fields A.J., Liebenberg E.C., Lim B.J., Larson P.E.Z., Gunduz-Demir C., Kazakia G.J., Krug R., Lotz J.C. (2018). Cartilage Endplate Thickness Variation Measured by Ultrashort Echo-Time MRI Is Associated with Adjacent Disc Degeneration. Spine.

[12] Grunhagen T., Shirazi-Adl A., Fairbank J.C.T., Urban J.P.G. (2011). Intervertebral Disk Nutrition: A Review of Factors Influencing Concentrations of Nutrients and Metabolites. Orthop. Clin. N. Am..

[13] Urban J.P.G., Smith S., Fairbank J.C.T. (2004). Nutrition of the intervertebral disc. Spine.

[14] Grant M.P., Epure L.M., Bokhari R., Roughley P., Antoniou J., Mwale F. (2016). Human cartilaginous endplate degeneration is induced by calcium and the extracellular calcium-sensing receptor in the intervertebral disc. Eur. Cells Mater..

[15] Wills C.R., Foata B., González Ballester M., Karppinen J., Noailly J. (2018). Theoretical explorations generate new hypotheses about the role of the cartilage endplate in early intervertebral disk degeneration. Front. Physiol..

[16] Iatridis J.C., Weidenbaum M., Setton L.A., Mow C.V. (1996). Is the nucleus pulposus a solid or a fluid? Mechanical behaviors of the nucleus pulposus of the human intervertebral disc. Spine.

[17] Sakai D., Grad S. (2015). Advancing the cellular and molecular therapy for intervertebral disc disease. Adv. Drug Deliv. Rev..

[18] Mwale F., Roughley P., Antoniou J., Alini M., Hollander A., Kirsch T., Stokes I. (2004). Distinction between the extracellular matrix of the nucleus pulposus and hyaline cartilage: A requisite for tissue engineering of intervertebral disc. Eur. Cells Mater..

[19] Umehara S., Tadano S., Abumi K., Katagiri K., Kaneda K., Ukai T. (1996). Effects of degeneration on the elastic modulus distribution in the lumbar intervertebral disc. Spine.

[20] Bron J.L., Koenderink G.H., Everts V., Smit T.H. (2009). Rheological characterization of the nucleus pulposus and dense collagen scaffolds intended for functional replacement. J. Orthop. Res..

[21] Iatridis J.C., Setton L.A., Weidenbaum M., Mow V.C. (1997). The viscoelastic behavior of the non-degenerate human lumbar nucleus pulposus in shear. J. Biomech..

[22] Race A., Broom N.D., Robertson P. (2000). Effect of loading rate and hydration on the mechanical properties of the disc. Spine.

[23] Cloyd J.M., Malhotra N.R., Weng L., Chen W., Mauck R.L., Elliott D.M. (2007). Material properties in unconfined compression of human nucleus pulposus, injectable hyaluronic acid-based hydrogels and tissue engineering scaffolds. Eur. Spine J..

[24] Iatridis J.C., Kumar S., Foster R.J., Weidenbaum M., Mow V.C. (1999). Shear mechanical properties of human lumbar annulus fibrosus. J. Orthop. Res..

[25] Adams M.A., Roughley P.J. (2006). What is intervertebral disc degeneration, and what causes it?. Spine.

[26] Cortes D.H., Elliott D.M., Shapiro I., Risbud M. (2014). The intervertebral disc: Overview of disc mechanics. The Intervertebral Disc: Molecular and Structural Studies of the Disc in Health and Disease.

[27] Maroudas A., Stockwell R.A., Nachemson A., Urban J. (1975). Factors involved in the nutrition of the human lumbar intervertebral disc: Cellularity and diffusion of glucose in vitro. J. Anat..

[28] Roughley P.J. (2004). Biology of intervertebral disc aging and degeneration: Involvement of the extracellular matrix. Spine.

[29] Risbud M.V., Schoepflin Z.R., Mwale F., Kandel R.A., Grad S., Iatridis J.C., Sakai D., Hoyland J.A. (2015). Defining the phenotype of young healthy nucleus pulposus cells: Recommendations of the spine research interest group at the 2014 annual ORS meeting. J. Orthop. Res..

[30] Bach F.C., Willems N., Penning L.C., Ito K., Meij B.P., Tryfonidou M.A. (2014). Potential regenerative treatment strategies for intervertebral disc degeneration in dogs. BMC Vet. Res..

[31] Neidlinger-Wilke C., Galbusera F., Pratsinis H., Mavrogonatou E., Mietsch A., Kletsas D., Wilke H.J. (2014). Mechanical loading of the intervertebral disc: From the macroscopic to the cellular level. Eur. Spine J..

[32] Marchand F., Ahmed A.M. (1990). Investigation of the laminate structure of lumbar disc anulus fibrosus. Spine.

[33] Disney C.M., Madi K., Bodey A.J., Lee P.D., Hoyland J.A., Sherratt M.J. (2017). Visualising the 3D microstructure of stained and native intervertebral discs using X-ray microtomography. Sci. Rep..

[34] Cassidy J.J., Hiltner A., Baer E. (1989). Hierarchical structure of the intervertebral disc. Connect. Tissue Res..

[35] Raza A., Michalek A.J. (2021). Radial trend in murine annulus fibrosus fiber orientation is best explained by vertebral growth. Eur. Spine J..

[36] Melrose J., Smith S.M., Appleyard R.C., Little C.B. (2008). Aggrecan, versican and type VI collagen are components of annular translamellar crossbridges in the intervertebral disc. Eur. Spine J..

[37] Smith L.J., Elliott D.M. (2011). Formation of lamellar cross bridges in the annulus fibrosus of the intervertebral disc is a consequence of vascular regression. Matrix Biol..

[38] Tavakoli J., Elliott D.M., Costi J.J. (2016). Structure and mechanical function of the inter-lamellar matrix of the annulus fibrosus in the disc. J. Orthop. Res..

[39] Disney C.M., Lee P.D., Hoyland J.A., Sherratt M.J., Bay B.K. (2018). A review of techniques for visualising soft tissue microstructure deformation and quantifying strain Ex Vivo. J. Microsc..

[40] Disney C.M., Eckersley A., McConnell J.C., Geng H., Bodey A.J., Hoyland J.A., Lee P.D., Sherratt M.J., Bay B.K. (2019). Synchrotron tomography of intervertebral disc deformation quantified by digital volume correlation reveals microstructural influence on strain patterns. Acta Biomater..

[41] Disney C.M., Mo J., Eckersley A., Bodey A.J., Hoyland J.A., Sherratt M.J., Pitsillides A.A., Lee P.D., Bay B.K. (2022). Regional variations in discrete collagen fibre mechanics within intact intervertebral disc resolved using synchrotron computed tomography and digital volume correlation. Acta Biomater..

[42] Pattappa G., Li Z., Peroglio M., Wismer N., Alini M., Grad S. (2012). Diversity of intervertebral disc cells: Phenotype and function. J. Anat..

[43] Singh K., Masuda K., Thonar E.J., An H.S., Cs-Szabo G. (2009). Age-related changes in the extracellular matrix of nucleus pulposus and anulus fibrosus of human intervertebral disc. Spine.

[44] Oegema T.R. (1993). Biochemistry of the Intervertebral Disc. Clin. Sports Med..

[45] Urban J., Maroudas A. (1980). The Chemistry of the Intervertebral Disc in Relation to its Physiological Function and Requirements. Clin. Rheum. Dis..

[46] Eyre D.R., Matsui Y., Wu J.J. (2002). Collagen polymorphisms of the intervertebral disc. Biochem. Soc. Trans..

[47] Sivan S., Hayes A., Wachtel E., Merkher Y., Owen S., Caterson B., Maroudas A., Roberts S. (2014). Biochemical composition and turnover of the extracellular matrix of the normal and degenerate intervertebral disc. Eur. Spine J..

[48] Nakai T., Sakai D., Nakamura Y., Nukaga T., Grad S., Li Z., Alini M., Chan D., Masuda K., Ando K. (2016). CD146 defines commitment of cultured annulus fibrosus cells to express a contractile phenotype. J. Orthop. Res..

[49] Clouet J., Grimandi G., Pot-Vaucel M., Masson M., Fellah H.B., Guigand L., Cherel Y., Bord E., Rannou F., Weiss P. (2009). Identification of phenotypic discriminating markers for intervertebral disc cells and articular chondrocytes. Rheumatology.

[50] van den Akker G.G.H., Eijssen L.M.T., Richardson S.M., Rhijn L.W.v., Hoyland J.A., Welting T.J.M., Voncken J.W. (2018). A Membranome-Centered Approach Defines Novel Biomarkers for Cellular Subtypes in the Intervertebral Disc. Cartilage.

[51] Sharabi M., Wade K., Haj-Ali R., Galbusera F., Wilke H. (2018). The Mechanical Role of Collagen Fibers in the Intervertebral Disc. Biomechanics of the Spine.

[52] Roberts S., Menage J., Urban J.P. (1989). Biochemical and structural properties of the cartilage end-plate and its relation to the intervertebral disc. Spine.

[53] Wu Y., Cisewski S.E., Sachs B.L., Pellegrini V.D., Kern M.J., Slate E.H., Yao H. (2015). The Region-dependent Biomechanical and Biochemical Properties of Bovine Cartilaginous Endplate. J. Biomech..

[54] Giers M.B., Munter B.T., Eyster K.J., Ide G.D., Newcomb A.G.U.S., Lehrman J.N., Belykh E., Byvaltsev V.A., Kelly B.P., Preul M.C. (2017). Biomechanical and Endplate Effects on Nutrient Transport in the Intervertebral Disc. World Neurosurg..

[55] Huang Y.C., Urban J.P.G., Luk K.D.K. (2014). Intervertebral disc regeneration: Do nutrients lead the way?. Nat. Rev. Rheumatol..

[56] Jackson A.R., Huang C.Y., Gua W.Y. (2011). Effect of endplate calcification and mechanical deformation on the distribution of glucose in intervertebral disc: A 3D finite element study. Comput. Methods Biomech. Biomed. Eng..

[57] St-Pierre J.P., Gan L., Wang J., Pilliar R.M., Grynpas M.D., Kandel R.A. (2012). The incorporation of a zone of calcified cartilage improves the interfacial shear strength between in vitro-formed cartilage and the underlying substrate. Acta Biomater..

[58] DeLucca J.F., Cortes D.H., Jacobs N.T., Vresilovic E.J., Duncan R.L., Elliott D.M. (2016). Human cartilage endplate permeability varies with degeneration and intervertebral disc site. J. Biomech..

[59] Naresh-Babu J., Neelima G., Reshma Begum S., Siva-Leela V. (2016). Diffusion characteristics of human annulus fibrosus—a study documenting the dependence of annulus fibrosus on end plate for diffusion. Spine J..

[60] Rajasekaran S., Venkatadass K., Naresh Babu J., Ganesh K., Shetty A.P. (2008). Pharmacological enhancement of disc diffusion and differentiation of healthy, ageing and degenerated discs: Results from in-vivo serial post-contrast MRI studies in 365 human lumbar discs. Eur. Spine J..

[61] Lakstins K., Arnold L., Gunsch G., Flanigan D., Khan S., Gadde N., Jones B., Agarwal G., Purmessur D. (2021). Characterization of the human intervertebral disc cartilage endplate at the molecular, cell, and tissue levels. J. Orthop. Res..

[62] Lakstins K., Arnold L., Gunsch G., Khan S., Moore S., Purmessur D. (2020). Characterization of bovine and canine animal model cartilage endplates and comparison to human cartilage endplate structure, matrix composition, and cell phenotype. JOR Spine.

[63] Smith S.M., Whitelock J.M., Iozzo R.V., Little C.B., Melrose J. (2009). Topographical variation in the distributions of versican, aggrecan and perlecan in the foetal human spine reflects their diverse functional roles in spinal development. Histochem. Cell Biol..

[64] Corallo D., Trapani V., Bonaldo P. (2015). The notochord: Structure and functions. Cell. Mol. Life Sci..

[65] Trapani V., Bonaldo P., Corallo D. (2017). Role of the ECM in notochord formation, function and disease. J. Cell Sci..

[66] Hwang P.Y., Chen J., Jing L., Hoffman B.D., Setton L.A. (2014). The Role of Extracellular Matrix Elasticity and Composition In Regulating the Nucleus Pulposus Cell Phenotype in the Intervertebral Disc: A Narrative Review. J. Biomech. Eng..

[67] Matta A., Erwin W.M. (2021). Current Status of the Instructional Cues Provided by Notochordal Cells in Novel Disc Repair Strategies. Int. J. Mol. Sci..

[68] Ward L., Rodrigues-Pinto R., Humphreys M., Hoyland J., Richardson S.M. (2018). Defining the phenotype of the human fetal notochordal cell: Implications for intervertebral disc regeneration. Osteoarthr. Cartil..

[69] Richardson S.M., Ludwinski F.E., Gnanalingham K.K., Atkinson R.A., Freemont A.J., Hoyland J.A. (2017). Notochordal and nucleus pulposus marker expression is maintained by sub-populations of adult human nucleus pulposus cells through aging and degeneration. Sci. Rep..

[70] van den Akker G., Welting T., Surtel D., Cremers A., Voncken W., van Rhijn L. (2012). Cell lines for the human intervertebral disc: Nucleus pulposus and annulus fibrosis. Osteoarthr. Cartil..

[71] van den Akker G.G.H., Surtel D.A.M., Cremers A., Rodrigues-Pinto R., Richardson S.M., Hoyland J.A., van Rhijn L.W., Welting T.J.M., Voncken J.W. (2014). Novel immortal human cell lines reveal subpopulations in the nucleus pulposus. Arthritis Res. Ther..

[72] Van Den Akker G.G.H., Surtel D.A.M., Cremers A., Richardson S.M., Hoyland J.A., Van Rhijn L.W., Voncken J.W., Welting T.J.M. (2016). Novel immortal cell lines support cellular heterogeneity in the human annulus fibrosus. PLoS ONE.

[73] van den Akker G.G.H., Koenders M.I., van de Loo F.A.J., van Lent P.L.E.M., Blaney Davidson E., van der Kraan P.M. (2017). Transcriptional profiling distinguishes inner and outer annulus fibrosus from nucleus pulposus in the bovine intervertebral disc. Eur. Spine J..

[74] Aguiar D.J., Johnson S.L., Oegema T.R. (1999). Notochordal cells interact with nucleus pulposus cells: Regulation of proteoglycan synthesis. Exp. Cell Res..

[75] Erwin W.M., Islam D., Inman R.D., Fehlings M.G., Tsui F.W.L. (2011). Notochordal cells protect nucleus pulposus cells from degradation and apoptosis: Implications for the mechanisms of intervertebral disc degeneration. Arthritis Res. Ther..

[76] De Vries S.A.H., Potier E., Van Doeselaar M., Meij B.P., Tryfonidou M.A., Ito K. (2015). Conditioned Medium Derived from Notochordal Cell-Rich Nucleus Pulposus Tissue Stimulates Matrix Production by Canine Nucleus Pulposus Cells and Bone Marrow-Derived Stromal Cells. Tissue Eng. Part A.

[77] Alkhatib B., Ban G.I., Williams S., Serra R. (2018). IVD Development: Nucleus pulposus development and sclerotome specification. Curr. Mol. Biol. Rep..

[78] Gilchrist C.L., Francisco A.T., Plopper G.E., Chen J., Setton L.A. (2011). Nucleus pulposus cell-matrix interactions with laminins. Eur. Cells Mater..

[79] Bridgen D.T., Gilchrist C.L., Richardson W.J., Isaacs R.E., Brown C.R., Yang K.L., Chen J., Setton L.A. (2013). Integrin-mediated interactions with extracellular matrix proteins for nucleus pulposus cells of the human intervertebral disc. J. Orthop. Res..

[80] Gilchrist C.L., Chen J., Richardson W.J., Loeser R.F., Setton L.A. (2007). Functional integrin subunits regulating cell-matrix interactions in the intervertebral disc. J. Orthop. Res..

[81] Barcellona M.N., Speer J.E., Fearing B.V., Jing L., Pathak A., Gupta M.C., Buchowski J.M., Kelly M., Setton L.A. (2020). Control of adhesive ligand density for modulation of nucleus pulposus cell phenotype. Biomaterials.

[82] Choi K.S., Harfe B.D. (2011). Hedgehog signaling is required for formation of the notochord sheath and patterning of nuclei pulposi within the intervertebral discs. Proc. Natl. Acad. Sci. USA.

[83] Rodrigues-Pinto R., Richardson S.M., Hoyland J.A. (2014). An understanding of intervertebral disc development, maturation and cell phenotype provides clues to direct cell-based tissue regeneration therapies for disc degeneration. Eur. Spine J..

[84] Hayes A.J., Lord M.S., Smith S.M., Smith M.M., Whitelock J.M., Weiss A.S., Melrose J. (2011). Colocalization in vivo and association in vitro of perlecan and elastin. Histochem. Cell Biol..

[85] Ghazanfari S., Werner A., Ghazanfari S., Weaver J.C., Smit T.H. (2018). Morphogenesis of aligned collagen fibers in the annulus fibrosus: Mammals versus avians. Biochem. Biophys. Res. Commun..

[86] Hayes A.J., Benjamin M., Ralphs J.R. (1999). Role of Actin Stress Fibres in the Development of the Intervertebral Disc: Cytoskeletal Control of Extracellular Matrix Assembly. Dev. Dyn..

[87] Rodrigues S.A., Thambyah A., Broom N.D. (2017). How maturity influences annulus-endplate integration in the ovine intervertebral disc: A micro- and ultra-structural study. J. Anat..

[88] Vergroesen P.P.A., Kingma I., Emanuel K.S., Hoogendoorn R.J.W., Welting T.J., van Royen B.J., van Dieën J.H., Smit T.H. (2015). Mechanics and biology in intervertebral disc degeneration: A vicious circle. Osteoarthr. Cartil..

[89] Sakai D., Nakamura Y., Nakai T., Mishima T., Kato S., Grad S., Alini M., Risbud M.V., Chan D., Cheah K.S.E. (2012). Exhaustion of nucleus pulposus progenitor cells with ageing and degeneration of the intervertebral disc. Nat. Commun..

[90] Risbud M.V., Shapiro I.M. (2014). Role of cytokines in intervertebral disc degeneration: Pain and disc content. Nat. Rev. Rheumatol..

[91] Le Maitre C.L., Freemont A.J., Hoyland J.A. (2005). The role of interleukin-1 in the pathogenesis of human intervertebral disc degeneration. Arthritis Res. Ther..

[92] Vo N.V., Hartman R.A., Yurube T., Jacobs L.J., Sowa G.A., Kang J.D. (2013). Expression and regulation of metalloproteinases and their inhibitors in intervertebral disc aging and degeneration. Spine J..

[93] Shiomi T., Lemaître V., D’Armiento J., Okada Y. (2010). Matrix metalloproteinases, a disintegrin and metalloproteinases, and a disintegrin and metalloproteinases with thrombospondin motifs in non-neoplastic diseases: Review Article. Pathol. Int..

[94] Jabłońska-Trypuć A., Matejczyk M., Rosochacki S. (2016). Matrix metalloproteinases (MMPs), the main extracellular matrix (ECM) enzymes in collagen degradation, as a target for anticancer drugs. J. Enzym. Inhib. Med. Chem..

[95] Fortelny N., Overall C.M., Pavlidis P., Freue G.V.C. (2017). Can we predict protein from mRNA levels?. Nature.

[96] Allegri M., Montella S., Salici F., Valente A., Marchesini M., Compagnone C., Baciarello M., Manferdini M.E., Fanelli G. (2016). Mechanisms of low back pain: A guide for diagnosis and therapy [version 2; peer review: 3 approved. F1000Research.

[97] Liu C., Yang M., Liu L., Zhang Y., Zhu Q., Huang C., Wang H., Zhang Y., Li H., Li C. (2020). Molecular basis of degenerative spinal disorders from a proteomic perspective (Review). Mol. Med. Rep..

[98] Brown S., Melrose J., Caterson B., Roughley P., Eisenstein S.M., Roberts S. (2012). A comparative evaluation of the small leucine-rich proteoglycans of pathological human intervertebral discs. Eur. Spine J..

[99] Craddock R.J., Hodson N.W., Ozols M., Shearer T., Hoyland J.A., Sherratt M.J. (2018). Extracellular matrix fragmentation in young, healthy cartilaginous tissues. Eur. Cells Mater..

[100] Patel K.P., Sandy J.D., Akeda K., Miyamoto K., Chujo T., An H.S., Masuda K. (2007). Aggrecanases and aggrecanase-generated fragments in the human intervertebral disc at early and advanced stages of disc degeneration. Spine.

[101] Sivan S.S., Wachtel E., Roughley P. (2014). Structure, function, aging and turnover of aggrecan in the intervertebral disc. Biochim. Biophys. Acta.

[102] O’Connell G.D., Guerin H.L., Elliott D.M. (2010). Theoretical and Uniaxial Experimental Evaluation of Human Annulus Fibrosus Degeneration. J. Biomech. Eng..

[103] Daly C., Ghosh P., Jenkin G., Oehme D., Goldschlager T. (2016). A Review of Animal Models of Intervertebral Disc Degeneration: Pathophysiology, Regeneration, and Translation to the Clinic. BioMed Res. Int..

[104] Hwang P.Y., Jing L., Chen J., Lim F.L., Tang R., Choi H., Cheung K.M., Risbud M.V., Gersbach C.A., Guilak F. (2016). N-cadherin is key to expression of the nucleus pulposus cell phenotype under selective substrate culture conditions. Sci. Rep..

[105] Rodrigues-Pinto R., Ward L., Humphreys M., Zeef L.A.H., Berry A., Hanley K.P., Hanley N., Richardson S.M., Hoyland J.A. (2018). Human notochordal cell transcriptome unveils potential regulators of cell function in the developing intervertebral disc. Sci. Rep..

[106] Risbud M.V., Shapiro I.M. (2012). Notochordal Cells in the Adult Intervertebral Disc: New Perspective on an Old Question. Crit. Rev. Eukaryot. Gene Expr..

[107] Li D., Zeng Q., Jiang Z., Ding L., Lu W., Bian M., Wu J. (2021). Induction of notochordal differentiation of bone marrow mesenchymal-derived stem cells via the stimulation of notochordal cell-rich nucleus pulposus tissue. Mol. Med. Rep..

[108] Li X.C., Wang M.S., Liu W., Zhong C.F., Deng G.B., Luo S.J., Huang C.M. (2018). Co-culturing nucleus pulposus mesenchymal stem cells with notochordal cell-rich nucleus pulposus explants attenuates tumor necrosis factor-α-induced senescence. Stem Cell Res. Ther..

[109] Diaz-Hernandez M.E., Khan N.M., Trochez C.M., Yoon T., Maye P., Presciutti S.M., Gibson G., Drissi H. (2020). Derivation of notochordal cells from human embryonic stem cells reveals unique regulatory networks by single cell-transcriptomics. J. Cell. Physiol..

[110] Rätsep T., Minajeva A., Asser T. (2013). Relationship between neovascularization and degenerative changes in herniated lumbar intervertebral discs. Eur. Spine J..

[111] Patil P., Niedernhofer L.J., Robbins P.D., Lee J., Sowa G., Vo N. (2018). Cellular senescence in intervertebral disc aging and degeneration. Curr. Mol. Biol. Rep..

[112] Khan A.N., Jacobsen H.E., Khan J., Filippi C.G., Levine M., Lehman R.A., Riew K.D., Lenke L.G., Chahine N.O. (2017). Inflammatory biomarkers of low back pain and disc degeneration: A review. Ann. N. Y. Acad. Sci..

[113] Pfirrmann C.W.A., Metzdorf A., Zanetti M., Hodler J., Boos N. (2001). Magnetic resonance classification of lumbar intervertebral disc degeneration. Spine.

[114] Cortes D.H., Jacobs N.T., DeLucca J.F., Elliott D.M. (2014). Elastic, permeability and swelling properties of human intervertebral disc tissues: A benchmark for tissue engineering. J. Biomech..

[115] Roberts S., Evans E.H., Kletsas D., Jaffray D.C., Eisenstein S.M. (2006). Senescence in Human Intervertebral Discs. Eur. Spine J..

[116] Horner H.A., Urban J.P.G. (2001). 2001 Volvo award winner in basic science studies: Effect of nutrient supply on the viability of cells from the nucleus pulposus of the intervertebral disc. Spine.

[117] Li F.C., Zhang N., Chen W.S., Chen Q.X. (2010). Endplate degeneration may be the origination of the vacuum phenomenon in intervertebral discs. Med. Hypotheses.

[118] Lu K., Li H.Y., Yang K., Wu J.L., Cai X.W., Zhou Y., Li C.Q. (2017). Exosomes as potential alternatives to stem cell therapy for intervertebral disc degeneration: In-vitro study on exosomes in interaction of nucleus pulposus cells and bone marrow mesenchymal stem cells. Stem Cell Res. Ther..

[119] Luo L., Jian X., Sun H., Qin J., Wang Y., Zhang J., Shen Z., Yang D., Li C., Zhao P. (2021). Cartilage endplate stem cells inhibit intervertebral disc degeneration by releasing exosomes to nucleus pulposus cells to activate Akt/autophagy. Stem Cells.

[120] Johnson W.E.B., Caterson B., Eisenstein S.M., Hynds D.L., Snow D.M., Roberts S. (2002). Human intervertebral disc aggrecan inhibits nerve growth in vitro. Arthritis Rheum..

[121] Kudelko M., Chen P., Tam V., Zhang Y., Kong O.Y., Sharma R., Au T.Y.K., To M.K.T., Cheah K.S.E., Chan W.C.W. (2021). PRIMUS: Comprehensive proteomics of mouse intervertebral discs that inform novel biology and relevance to human disease modelling. Matrix Biol. Plus.

[122] Wang H., Wang D., Luo B., Wang D., Jia H., Peng P., Shang Q., Mao J., Gao C., Peng Y. (2022). Decoding the annulus fibrosus cell atlas by scRNA-seq to develop an inducible composite hydrogel: A novel strategy for disc reconstruction. Bioact. Mater..

[123] Li K., Kapper D., Mondal S., Lufkin T., Kraus P. (2019). Quantitative Single-Cell Transcript Assessment of Biomarkers Supports Cellular Heterogeneity in the Bovine IVD. Vet. Sci..

[124] Veras M.A., McCann M.R., Tenn N.A., Séguin C.A. (2020). Transcriptional profiling of the murine intervertebral disc and age-associated changes in the nucleus pulposus. Connect. Tissue Res..

[125] Caldeira J., Santa C., Osório H., Molinos M., Manadas B., Goncalves R., Barbosa M. (2017). Matrisome Profiling during Intervertebral Disc Development and Ageing. Sci. Rep..

[126] Smolders L.A., Meij B.P., Onis D., Riemers F.M., Bergknut N., Wubbolts R., Grinwis G.C.M., Houweling M., Groot Koerkamp M.J.A., van Leenen D. (2013). Gene expression profiling of early intervertebral disc degeneration reveals a down-regulation of canonical Wnt signaling and caveolin-1 expression: Implications for development of regenerative strategies. Arthritis Res. Ther..

[127] Minogue B.M., Richardson S.M., Zeef L.A.H., Freemont A.J., Hoyland J.A. (2010). Transcriptional profiling of bovine intervertebral disc cells: Implications for identification of normal and degenerate human intervertebral disc cell phenotypes. Arthritis Res. Ther..

[128] Eckersley A., Ozols M., Chen P., Tam V., Hoyland J.A., Trafford A., Chan D., Sherratt M.J. (2021). Peptide Location Fingerprinting Reveals Tissue Region-Specific Differences in Protein Structures in an Ageing Human Organ. Int. J. Mol. Sci..

[129] Minogue B.M., Richardson S.M., Zeef L.A.H., Freemont A.J., Hoyland J.A. (2010). Characterization of the human nucleus pulposus cell phenotype and evaluation of novel marker gene expression to define adult stem cell differentiation. Arthritis Rheum..

[130] Fernandes L.M., Khan N.M., Trochez C.M., Duan M., Diaz-Hernandez M.E., Presciutti S.M., Gibson G., Drissi H. (2020). Single-cell RNA-seq identifies unique transcriptional landscapes of human nucleus pulposus and annulus fibrosus cells. Sci. Rep..

[131] Gan Y., He J., Zhu J., Xu Z., Wang Z., Yan J., Hu O., Bai Z., Chen L., Xie Y. (2021). Spatially defined single-cell transcriptional profiling characterizes diverse chondrocyte subtypes and nucleus pulposus progenitors in human intervertebral discs. Bone Res..

[132] Barcellona M.N., Speer J.E., Jing L., Gupta M.C., Buchowski J.M., Kelly M.P., Setton L.A., Lopata S. (2021). Engineered Peptide-Functionalized Hydrogels Modulate the RNA Transcriptome of Human Nucleus Pulposus Cells In Vitro. bioRxiv.

[133] Riester S.M., Lin Y., Wang W., Cong L., Mohamed Ali A.M., Peck S.H., Smith L.J., Currier B.L., Clark M., Huddleston P. (2018). RNA sequencing identifies gene regulatory networks controlling extracellular matrix synthesis in intervertebral disk tissues. J. Orthop. Res. Off. Publ. Orthop. Res. Soc..

[134] Rajasekaran S., Soundararajan D.C.R., Tangavel C., Sri Vijay Anand K.S., Nayagam S.M., Matchado M.S., Muthurajan R., Shetty A.P., Kanna R.M., Dharmalingam K. (2020). Proteomic Signature of Nucleus Pulposus in Fetal Intervertebral Disc. Asian Spine J..

[135] Qiu C., Wu X., Bian J., Ma X., Zhang G., Guo Z., Wang Y., Ci Y., Wang Q., Xiang H. (2020). Differential proteomic analysis of fetal and geriatric lumbar nucleus pulposus: Immunoinflammation and age-related intervertebral disc degeneration. BMC Musculoskelet. Disord..

[136] Rajasekaran S., Thangavel C., Djuric N., Raveendran M., Soundararajan D.C.R., Nayagam S.M., Matchado M.S., Sri Vijay Anand K.S., Venkateshwaran K. (2021). Profiling extra cellular matrix associated proteome of human fetal nucleus pulposus in search for regenerative targets. Sci. Rep..

[137] Ye D., Liang W., Dai L., Zhou L., Yao Y., Zhong X., Chen H., Xu J. (2015). Comparative and quantitative proteomic analysis of normal and degenerated human annulus fibrosus cells. Clin. Exp. Pharmacol. Physiol..

[138] Önnerfjord P., Khabut A., Reinholt F.P., Svensson O., Heinegård D. (2012). Quantitative Proteomic Analysis of Eight Cartilaginous Tissues Reveals Characteristic Differences as well as Similarities between Subgroups. J. Biol. Chem..

[139] Yee A., Lam M.P.Y., Tam V., Chan W.C.W., Chu I.K., Cheah K.S.E., Cheung K.M.C., Chan D. (2016). Fibrotic-like changes in degenerate human intervertebral discs revealed by quantitative proteomic analysis. Osteoarthr. Cartil..

[140] Rajasekaran S., Tangavel C., Sri Vijay Anand K.S., Soundararajan D.C.R., Nayagam S.M., Matchado M.S., Raveendran M., Shetty A.P., Kanna R.M., Dharmalingam K. (2020). Inflammaging determines health and disease in lumbar discs-evidence from differing proteomic signatures of healthy, aging, and degenerating discs. Spine J..

[141] Sarath Babu N., Krishnan S., Brahmendra Swamy C.V., Venkata Subbaiah G.P., Gurava Reddy A.V., Idris M.M. (2016). Quantitative proteomic analysis of normal and degenerated human intervertebral disc. Spine J..

[142] Wangler S., Kamali A., Wapp C., Wuertz-Kozak K., Häckel S., Fortes C., Benneker L.M., Haglund L., Richards R.G., Alini M. (2021). Uncovering the secretome of mesenchymal stromal cells exposed to healthy, traumatic, and degenerative intervertebral discs: A proteomic analysis. Stem Cell Res. Ther..

[143] Radek M., Pacholczyk-Sienicka B., Jankowski S., Albrecht Ł., Grodzka M., Depta A., Radek A. (2016). Assessing the correlation between the degree of disc degeneration on the Pfirrmann scale and the metabolites identified in HR-MAS NMR spectroscopy. Magn. Reson. Imaging.

[144] National Guideline Centre (UK) (2016). Low Back Pain and Sciatica in over 16s: Assessment and Management.

[145] Dagenais S., Caro J., Haldeman S. (2008). A systematic review of low back pain cost of illness studies in the United States and internationally. Spine J..

[146] Walker B.F. (2000). The prevalence of low back pain: A systematic review of the literature from 1966 to 1998. J. Spinal Disord..

[147] Vos T., Flaxman A.D., Naghavi M., Lozano R., Michaud C., Ezzati M., Shibuya K., Salomon J.A., Abdalla S., Aboyans V. (2012). Years lived with disability (YLDs) for 1160 sequelae of 289 diseases and injuries 1990–2010: A systematic analysis for the Global Burden of Disease Study 2010. Lancet.

[148] Foster N.E., Anema J.R., Cherkin D., Chou R., Cohen S.P., Gross D.P., Ferreira P.H., Fritz J.M., Koes B.W., Peul W. (2018). Prevention and treatment of low back pain: Evidence, challenges, and promising directions. Lancet.

[149] Zigler J., Gornet M.F., Ferko N., Cameron C., Schranck F.W., Patel L. (2017). Comparison of Lumbar Total Disc Replacement with Surgical Spinal Fusion for the Treatment of Single-Level Degenerative Disc Disease: A Meta-Analysis of 5-Year Outcomes from Randomized Controlled Trials. Glob. Spine J..

[150] Nguyen T.H., Randolph D.C., Talmage J., Succop P., Travis R. (2011). Long-term outcomes of lumbar fusion among workers’ compensation subjects: A historical cohort study. Spine.

[151] Frost B.A., Camarero-Espinosa S., Johan Foster E. (2019). Materials for the spine: Anatomy, problems, and solutions. Materials.

[152] Ruan D., He Q., Ding Y., Hou L., Li J., Luk K.D.K. (2007). Intervertebral disc transplantation in the treatment of degenerative spine disease: A preliminary study. Lancet.

[153] Tarpada S.P., Morris M.T., Burton D.A. (2017). Spinal fusion surgery: A historical perspective. J. Orthop..

[154] Meng B., Bunch J., Burton D., Wang J. (2021). Lumbar interbody fusion: Recent advances in surgical techniques and bone healing strategies. Eur. Spine J..

[155] Iatridis J.C., Nicoll S.B., Michalek A.J., Walter B.A., Gupta M.S. (2013). Role of biomechanics in intervertebral disc degeneration and regenerative therapies: What needs repairing in the disc and what are promising biomaterials for its repair?. Spine J..

[156] Sloan S.R., Lintz M., Hussain I., Hartl R., Bonassar L.J. (2018). Biologic Annulus Fibrosus Repair: A Review of Preclinical in Vivo Investigations. Tissue Eng. Part B Rev..

[157] Hom W.W., Tschopp M., Lin H.A., Nasser P., Laudier D.M., Hecht A.C., Nicoll S.B., Iatridis J.C. (2019). Composite biomaterial repair strategy to restore biomechanical function and reduce herniation risk in an ex vivo large animal model of intervertebral disc herniation with varying injury severity. PLoS ONE.

[158] Iatridis J.C. (2017). Structural and Functional Repair of the Annulus Fibrosus. Glob. Spine J..

[159] Tuakli-Wosornu Y.A., Terry A., Boachie-Adjei K., Harrison J.R., Gribbin C.K., LaSalle E.E., Nguyen J.T., Solomon J.L., Lutz G.E. (2016). Lumbar Intradiskal Platelet-Rich Plasma (PRP) Injections: A Prospective, Double-Blind, Randomized Controlled Study. Phys. Med. Rehabil..

[160] Comella K., Silbert R., Parlo M. (2017). Effects of the intradiscal implantation of stromal vascular fraction plus platelet rich plasma in patients with degenerative disc disease. J. Transl. Med..

[161] Pirvu T.N., Schroeder J.E., Peroglio M., Verrier S., Kaplan L., Richards R.G., Alini M., Grad S. (2014). Platelet-rich plasma induces annulus fibrosus cell proliferation and matrix production. Eur. Spine J..

[162] Chen W.H., Liu H.Y., Lo W.C., Wu S.C., Chi C.H., Chang H.Y., Hsiao S.H., Wu C.H., Chiu W.T., Chen B.J. (2009). Intervertebral disc regeneration in an ex vivo culture system using mesenchymal stem cells and platelet-rich plasma. Biomaterials.

[163] Gantenbein B., Illien-Jünger S., Chan S.C.W., Walser J., Haglund L., Ferguson S.J., Iatridis J.C., Grad S. (2015). Organ culture bioreactors-platforms to study human intervertebral disc degeneration and regenerative therapy. Curr. Stem Cell Res. Ther..

[164] Clouet J., Fusellier M., Camus A., Le Visage C., Guicheux J. (2019). Intervertebral disc regeneration: From cell therapy to the development of novel bioinspired endogenous repair strategies. Adv. Drug Deliv. Rev..

[165] Roh E.J., Darai A., Kyung J.W., Choi H., Kwon S.Y., Bhujel B., Kim K.T., Han I. (2021). Genetic Therapy for Intervertebral Disc Degeneration. Int. J. Mol. Sci..

[166] Farhang N., Ginley-Hidinger M., Berrett K.C., Gertz J., Lawrence B., Bowles R.D. (2019). Lentiviral CRISPR Epigenome Editing of Inflammatory Receptors as a Gene Therapy Strategy for Disc Degeneration. Hum. Gene Ther..

[167] Liu Z., Zheng Z., Qi J., Wang J., Zhou Q., Hu F., Liang J., Li C., Zhang W., Zhang X. (2018). CD24 identifies nucleus pulposus progenitors/notochordal cells for disc regeneration. J. Biol. Eng..

[168] Williams R.J., Tryfonidou M.A., Snuggs J.W., Le Maitre C.L. (2021). Cell sources proposed for nucleus pulposus regeneration. JOR Spine.

[169] Gay M.H.P., Mehrkens A., Rittmann M., Haug M., Barbero A., Martin I., Schaeren S. (2019). Nose to back: Compatibility of nasal chondrocytes with environmental conditions mimicking a degenerated intervertebral disc. Eur. Cells Mater..

[170] Kuh S.U., Zhu Y., Li J., Tsai K.J., Fei Q., Hutton W.C., Yoon T.S. (2009). A comparison of three cell types as potential candidates for intervertebral disc therapy: Annulus fibrosus cells, chondrocytes, and bone marrow derived cells. Jt. Bone Spine.

[171] Sakai D., Andersson G.B.J. (2015). Stem cell therapy for intervertebral disc regeneration: Obstacles and solutions. Nat. Rev. Rheumatol..

[172] Mobasheri A., Richardson S.M. (2020). Cell and Gene Therapy for Spine Regeneration: Mammalian Protein Production Platforms for Overproduction of Therapeutic Proteins and Growth Factors. Neurosurg. Clin. N. Am..

[173] Melrose J. (2016). Strategies in regenerative medicine for intervertebral disc repair using mesenchymal stem cells and bioscaffolds. Regen. Med..

[174] Clarke L.E., McConnell J.C., Sherratt M.J., Derby B., Richardson S.M., Hoyland J.A. (2014). Growth differentiation factor 6 and transforming growth factor-beta differentially mediate mesenchymal stem cell differentiation, composition, and micromechanical properties of nucleus pulposus constructs. Arthritis Res. Ther..

[175] Chujo T., An H.S., Akeda K., Miyamoto K., Muehleman C., Attawia M., Andersson G., Masuda K. (2006). Effects of growth differentiation factor-5 on the intervertebral disc—In vitro bovine study and in vivo rabbit disc degeneration model study. Spine.

[176] Loibl M., Wuertz-Kozak K., Vadala G., Lang S., Fairbank J., Urban J.P. (2019). Controversies in regenerative medicine: Should intervertebral disc degeneration be treated with mesenchymal stem cells?. JOR Spine.

[177] Migliorini F., Rath B., Tingart M., Baroncini A., Quack V., Eschweiler J. (2018). Autogenic mesenchymal stem cells for intervertebral disc regeneration. Int. Orthop..

[178] Stoyanov J.V., Gantenbein-Ritter B., Bertolo A., Aebli N., Baur M., Alini M., Grad S. (2011). Role of hypoxia and growth and differentiation factor-5 on differentiation of human mesenchymal stem cells towards intervertebral nucleus pulposus-like cells. Eur. Cells Mater..

[179] Zeckser J., Wolff M., Tucker J., Goodwin J. (2016). Multipotent Mesenchymal Stem Cell Treatment for Discogenic Low Back Pain and Disc Degeneration. Stem Cells Int..

[180] Binch A.L.A., Fitzgerald J.C., Growney E.A., Barry F. (2021). Cell-based strategies for IVD repair: Clinical progress and translational obstacles. Nat. Rev. Rheumatol..

[181] Kennon J.C., Awad M.E., Chutkan N., Devine J., Fulzele S. (2018). Current insights on use of growth factors as therapy for Intervertebral Disc Degeneration. Biomol. Concepts.

[182] Ho G., Leung V.Y.L., Cheung K.M.C., Chan D. (2008). Effect of severity of intervertebral disc injury on mesenchymal stem cell-based regeneration. Connect. Tissue Res..

[183] Wuertz K., Godburn K., Neidlinger-Wilke C., Urban J., Iatridis J.C. (2008). Behavior of Mesenchymal Stem Cells in the Chemical Microenvironment of the Intervertebral Disc. Spine.

[184] Kluba T., Niemeyer T., Gaissmaier C., Gründer T. (2005). Human anulus fibrosis and nucleus pulposus cells of the intervertebral disc: Effect of degeneration and culture system on cell phenotype. Spine.

[185] Speer J.E., Barcellona M.N., Lu M.Y., Zha Z., Jing L., Gupta M.C., Buchowski J.M., Kelly M.P., Setton L.A. (2021). Development of a library of laminin-mimetic peptide hydrogels for control of nucleus pulposus cell behaviors. J. Tissue Eng..

[186] Tan X., Jain E., Barcellona M.N., Morris E., Neal S., Gupta M.C., Buchowski J.M., Kelly M., Setton L.A., Huebsch N. (2021). Integrin and syndecan binding peptide-conjugated alginate hydrogel for modulation of nucleus pulposus cell phenotype. Biomaterials.

[187] Zhou X., Tao Y., Chen E., Wang J., Fang W., Zhao T., Liang C., Li F., Chen Q. (2018). Genipin-cross-linked type II collagen scaffold promotes the differentiation of adipose-derived stem cells into nucleus pulposus-like cells. J. Biomed. Mater. Res. Part A.

[188] Chen P., Ning L., Qiu P., Mo J., Mei S., Xia C., Zhang J., Lin X., Fan S. (2019). Photo-crosslinked gelatin-hyaluronic acid methacrylate hydrogel-committed nucleus pulposus-like differentiation of adipose stromal cells for intervertebral disc repair. J. Tissue Eng. Regen. Med..

[189] Wang J., Tao Y., Zhou X., Li H., Liang C., Li F., Chen Q.X. (2016). The potential of chondrogenic pre-differentiation of adipose-derived mesenchymal stem cells for regeneration in harsh nucleus pulposus microenvironment. Exp. Biol. Med..

[190] Huang B., Zhuang Y., Li C.Q., Liu L.T., Zhou Y. (2011). Regeneration of the intervertebral disc with nucleus pulposus cell-seeded collagen II/hyaluronan/chondroitin-6-sulfate tri-copolymer constructs in a rabbit disc degeneration model. Spine.

[191] Grunert P., Borde B.H., Towne S.B., Moriguchi Y., Hudson K.D., Bonassar L.J., Härtl R. (2015). Riboflavin crosslinked high-density collagen gel for the repair of annular defects in intervertebral discs: An in vivo study. Acta Biomater..

[192] Borde B., Grunert P., Härtl R., Bonassar L.J. (2015). Injectable, high-density collagen gels for annulus fibrosus repair: An in vitro rat tail model. J. Biomed. Mater. Res. Part A.

[193] Guillaume O., Naqvi S.M., Lennon K., Buckley C.T. (2015). Enhancing cell migration in shape-memory alginate-collagen composite scaffolds: In vitro and ex vivo assessment for intervertebral disc repair. J. Biomater. Appl..

[194] Du J., Long R.G., Nakai T., Sakai D., Benneker L.M., Zhou G., Li B., Eglin D., Iatridis J.C., Alini M. (2020). Functional cell phenotype induction with TGF-β1 and collagen-polyurethane scaffold for annulus fibrosus rupture repair. Eur. Cells Mater..

[195] Hussain I., Sloan S.R., Wipplinger C., Navarro-Ramirez R., Zubkov M., Kim E., Kirnaz S., Bonassar L.J., Härtl R. (2019). Mesenchymal Stem Cell-Seeded High-Density Collagen Gel for Annular Repair: 6-Week Results from in Vivo Sheep Models. Neurosurgery.

[196] Liu C., Jin Z., Ge X., Zhang Y., Xu H. (2019). Decellularized Annulus Fibrosus Matrix/Chitosan Hybrid Hydrogels with Basic Fibroblast Growth Factor for Annulus Fibrosus Tissue Engineering. Tissue Eng. Part A.

[197] Kilmer C.E., Battistoni C.M., Cox A., Breur G.J., Panitch A., Liu J.C. (2020). Collagen Type I and II Blend Hydrogel with Autologous Mesenchymal Stem Cells as a Scaffold for Articular Cartilage Defect Repair. ACS Biomater. Sci. Eng..

[198] Kilmer C.E., Walimbe T., Panitch A., Liu J.C. (2022). Incorporation of a Collagen-Binding Chondroitin Sulfate Molecule to a Collagen Type I and II Blend Hydrogel for Cartilage Tissue Engineering. ACS Biomater. Sci. Eng..

[199] Walimbe T., Calve S., Panitch A., Sivasankar M.P. (2019). Incorporation of types I and III collagen in tunable hyaluronan hydrogels for vocal fold tissue engineering. Acta Biomater..

[200] Walimbe T., Panitch A. (2020). Best of Both Hydrogel Worlds: Harnessing Bioactivity and Tunability by Incorporating Glycosaminoglycans in Collagen Hydrogels. Bioengineering.

[201] Sharma S., Panitch A., Neu C.P. (2013). Incorporation of an aggrecan mimic prevents proteolytic degradation of anisotropic cartilage analogs. Acta Biomater..

[202] Sarkar S., Moorehead C., Prudnikova K., Schauer C.L., Penn L.S., Marcolongo M. (2017). Synthesis of macromolecular mimics of small leucine-rich proteoglycans with a poly(ethylene glycol) core and chondroitin sulphate bristles. Carbohydr. Polym..

[203] Prudnikova K., Yucha R.W., Patel P., Kriete A.S., Han L., Penn L.S., Marcolongo M.S. (2017). Biomimetic Proteoglycans Mimic Macromolecular Architecture and Water Uptake of Natural Proteoglycans. Biomacromolecules.

[204] Sarkar S., Lightfoot-Vidal S.E., Schauer C.L., Vresilovic E., Marcolongo M. (2012). Terminal-end functionalization of chondroitin sulfate for the synthesis of biomimetic proteoglycans. Carbohydr. Polym..

[205] Bernhard J.C., Panitch A. (2012). Synthesis and characterization of an aggrecan mimic. Acta Biomater..

[206] Sharma S., Lee A., Choi K., Kim K., Youn I., Trippel S.B., Panitch A. (2013). Biomimetic Aggrecan Reduces Cartilage Extracellular Matrix from Degradation and Lowers Catabolic Activity in Ex Vivo and In Vivo Models. Macromol. Biosci..

[207] Phillips E.R., Haislup B.D., Bertha N., Lefchak M., Sincavage J., Prudnikova K., Shallop B., Mulcahey M.K., Marcolongo M.S. (2019). Biomimetic proteoglycans diffuse throughout articular cartilage and localize within the pericellular matrix. J. Biomed. Mater. Res. Part A.

[208] Wu J., Shimmon S., Paton S., Daly C., Goldschlager T., Gronthos S., Zannettino A.C.W., Ghosh P. (2017). Pentosan polysulfate binds to STRO-1+ mesenchymal progenitor cells, is internalized, and modifies gene expression: A novel approach of pre-programing stem cells for therapeutic application requiring their chondrogenesis. Stem Cell Res. Ther..

[209] Hayes A.J., Melrose J. (2019). Glycosaminoglycan and Proteoglycan Biotherapeutics in Articular Cartilage Protection and Repair Strategies: Novel Approaches to Visco-supplementation in Orthobiologics. Adv. Ther..

[210] Farrugia B.L., Lord M.S., Whitelock J.M., Melrose J. (2018). Harnessing chondroitin sulphate in composite scaffolds to direct progenitor and stem cell function for tissue repair. Biomater. Sci..

[211] Prudnikova K., Lightfoot Vidal S.E., Sarkar S., Yu T., Yucha R.W., Ganesh N., Penn L.S., Han L., Schauer C.L., Vresilovic E.J. (2018). Aggrecan-like biomimetic proteoglycans (BPGs) composed of natural chondroitin sulfate bristles grafted onto a poly(acrylic acid) core for molecular engineering of the extracellular matrix. Acta Biomater..

[212] Madende D., Prudnikova K., Lightfoot S., Vresilovic E., Marcolongo M. New biomimetic aggrecan for treatment of intervertebral disc degeneration. Proceedings of the 2012 38th Annual Northeast Bioengineering Conference (NEBEC).

[213] Smith M.M., Hayes A.J., Melrose J. (2022). Pentosan Polysulfate, a Semisynthetic Heparinoid Disease-Modifying Osteoarthritic Drug with Roles in Intervertebral Disc Repair Biology Emulating the Stem Cell Instructive and Tissue Reparative Properties of Heparan Sulfate. Stem Cells Dev..

[214] Sivan S.S., Roberts S., Urban J.P.G., Menage J., Bramhill J., Campbell D., Franklin V.J., Lydon F., Merkher Y., Maroudas A. (2014). Injectable hydrogels with high fixed charge density and swelling pressure for nucleus pulposus repair: Biomimetic glycosaminoglycan analogues. Acta Biomater..

[215] Knani D., Eylon M., Sivan S.S. (2020). Molecular modeling study of the swelling of glycosaminoglycan-analog biomimetics for intervertebral disc repair. Polym. Adv. Technol..

[216] Farrugia B., Hayes A.J., Melrose J., Götte M., Forsberg-Nilsson K. (2021). Use of Chondroitin Sulphate to Aid In Vitro Stem Cell Differentiation. Proteoglycans in Stem Cells: From Development to Cancer.

[217] Place L.W., Kelly S.M., Kipper M.J. (2014). Synthesis and characterization of proteoglycan-mimetic graft copolymers with tunable glycosaminoglycan density. Biomacromolecules.

[218] Vadalà G., Russo F., Musumeci M., D’Este M., Cattani C., Catanzaro G., Tirindelli M.C., Lazzari L., Alini M., Giordano R. (2017). Clinically relevant hydrogel-based on hyaluronic acid and platelet rich plasma as a carrier for mesenchymal stem cells: Rheological and biological characterization. J. Orthop. Res..

[219] Pati F., Jang J., Ha D.H., Won Kim S., Rhie J.W., Shim J.H., Kim D.H., Cho D.W. (2014). Printing three-dimensional tissue analogues with decellularized extracellular matrix bioink. Nat. Commun..

[220] Fiordalisi M., Silva A.J., Barbosa M., Goncalves R., Caldeira J. (2020). Decellularised Scaffolds for Intervertebral Disc Regeneration. Trends Biotechnol..

[221] Hoshiba T., Chen G., Endo C., Maruyama H., Wakui M., Nemoto E., Kawazoe N., Tanaka M. (2016). Decellularized Extracellular Matrix as an In Vitro Model to Study the Comprehensive Roles of the ECM in Stem Cell Differentiation. Stem Cells Int..

[222] Penolazzi L., Pozzobon M., Bergamin L.S., D’Agostino S., Francescato R., Bonaccorsi G., De Bonis P., Cavallo M., Lambertini E., Piva R. (2020). Extracellular Matrix from Decellularized Wharton’s Jelly Improves the Behavior of Cells from Degenerated Intervertebral Disc. Front. Bioeng. Biotechnol..

[223] Kim B.S., Kim H., Gao G., Jang J., Cho D.W. (2017). Decellularized extracellular matrix: A step towards the next generation source for bioink manufacturing. Biofabrication.

[224] Kim B.S., Das S., Jang J., Cho D.W. (2020). Decellularized Extracellular Matrix-based Bioinks for Engineering Tissue- and Organ-specific Microenvironments. Chem. Rev..

[225] Pina S., Ribeiro V.P., Marques C.F., Maia F.R., Silva T.H., Reis R.L., Oliveira J.M. (2019). Scaffolding Strategies for Tissue Engineering and Regenerative Medicine Applications. Materials.

[226] Taylor D.A., Sampaio L.C., Ferdous Z., Gobin A.S., Taite L.J. (2018). Decellularized matrices in regenerative medicine. Acta Biomater..

[227] Parmaksiz M., Dogan A., Odabas S., Elcin A.E., Elcin Y.M. (2016). Clinical applications of decellularized extracellular matrices for tissue engineering and regenerative medicine. Biomed. Mater..

[228] Chae S., Cho D.W. (2022). Three-dimensional bioprinting with decellularized extracellular matrix-based bioinks in translational regenerative medicine. MRS Bull..

[229] Tsaryk R., Gloria A., Russo T., Anspach L., De Santis R., Ghanaati S., Unger R.E., Ambrosio L., Kirkpatrick C.J. (2015). Collagen-low molecular weight hyaluronic acid semi-interpenetrating network loaded with gelatin microspheres for cell and growth factor delivery for nucleus pulposus regeneration. Acta Biomater..

[230] Collin E.C., Grad S., Zeugolis D.I., Vinatier C.S., Clouet J.R., Guicheux J.J., Weiss P., Alini M., Pandit A.S. (2011). An injectable vehicle for nucleus pulposus cell-based therapy. Biomaterials.

[231] Fontana G., Thomas D., Collin E., Pandit A., Fontana G., Thomas D., Collin E., Pandit A. (2014). Microgel Microenvironment Primes Adipose-Derived Stem Cells Towards an NP Cells-Like Phenotype. Adv. Healthc. Mater..

[232] Srivastava A., Isa I.L.M., Rooney P., Pandit A. (2017). Bioengineered three-dimensional diseased intervertebral disc model revealed inflammatory crosstalk. Biomaterials.

[233] Borrelli C., Buckley C.T. (2020). Injectable Disc-Derived ECM Hydrogel Functionalised with Chondroitin Sulfate for Intervertebral Disc Regeneration. Acta Biomater..

[234] Francisco A.T., Mancino R.J., Bowles R.D., Brunger J.M., Tainter D.M., Chen Y.T., Richardson W.J., Guilak F., Setton L.A. (2013). Injectable laminin-functionalized hydrogel for nucleus pulposus regeneration. Biomaterials.

[235] Peroglio M., Eglin D., Benneker L.M., Alini M., Grad S. (2013). Thermoreversible hyaluronan-based hydrogel supports in vitro and ex vivo disc-like differentiation of human mesenchymal stem cells. Spine J..

[236] Jeong C.G., Francisco A.T., Niu Z., Mancino R.L., Craig S.L., Setton L.A. (2014). Screening of hyaluronic acid-poly(ethylene glycol) composite hydrogels to support intervertebral disc cell biosynthesis using artificial neural network analysis. Acta Biomater..

[237] Isa I.L.M., Srivastava A., Tiernan D., Owens P., Rooney P., Dockery P., Pandit A. (2015). Hyaluronic acid based hydrogels attenuate inflammatory receptors and neurotrophins in interleukin-1β induced inflammation model of nucleus pulposus cells. Biomacromolecules.

[238] Mohd Isa I.L., Abbah S.A., Kilcoyne M., Sakai D., Dockery P., Finn D.P., Pandit A. (2018). Implantation of hyaluronic acid hydrogel prevents the pain phenotype in a rat model of intervertebral disc injury. Sci. Adv..

[239] Fuller E.S., Shu C., Smith M.M., Little C.B., Melrose J. (2018). Hyaluronan oligosaccharides stimulate matrix metalloproteinase and anabolic gene expression in vitro by intervertebral disc cells and annular repair in vivo. J. Tissue Eng. Regen. Med..

[240] Frith J.E., Menzies D.J., Cameron A.R., Ghosh P., Whitehead D.L., Gronthos S., Zannettino A.C.W., Cooper-White J.J. (2014). Effects of bound versus soluble pentosan polysulphate in PEG/HA-based hydrogels tailored for intervertebral disc regeneration. Biomaterials.

[241] Oehme D., Ghosh P., Shimmon S., Wu J., McDonald C., Troupis J.M., Goldschlager T., Rosenfeld J.V., Jenkin G. (2014). Mesenchymal progenitor cells combined with pentosan polysulfate mediating disc regeneration at the time of microdiscectomy: A preliminary study in an ovine model: Laboratory investigation. J. Neurosurg. Spine.

[242] Costa J.B., Silva-Correia J., Ribeiro V.P., da Silva Morais A., Oliveira J.M., Reis R.L. (2019). Engineering patient-specific bioprinted constructs for treatment of degenerated intervertebral disc. Mater. Today Commun..

[243] Bello A.B., Kim Y., Park S., Muttigi M.S., Kim J., Park H., Lee S. (2021). Matrilin3/TGFβ3 gelatin microparticles promote chondrogenesis, prevent hypertrophy, and induce paracrine release in MSC spheroid for disc regeneration. NPJ Regen. Med..

[244] Bridgen D.T., Fearing B.V., Jing L., Sanchez-Adams J., Cohan M.C., Guilak F., Chen J., Setton L.A. (2017). Regulation of human nucleus pulposus cells by peptide-coupled substrates. Acta Biomater..

[245] Barcellona M.N., Speer J.E., Jing L., Patil D.S., Gupta M.C., Buchowski J.M., Setton L.A. (2021). Bioactive in situ crosslinkable polymer-peptide hydrogel for cell delivery to the intervertebral disc in a rat model. Acta Biomater..

[246] Guilak F., Hayes A.J., Melrose J. (2021). Perlecan in pericellular mechanosensory cell-matrix communication, extracellular matrix stabilisation and mechanoregulation of load-bearing connective tissues. Int. J. Mol. Sci..

[247] Smith S.M., Melrose J. (2019). Type XI collagen-perlecan-HS interactions stabilise the pericellular matrix of annulus fibrosus cells and chondrocytes providing matrix stabilisation and homeostasis. J. Mol. Histol..

[248] Hayes A.J., Melrose J. (2021). What Are the Potential Roles of Nuclear Perlecan and Other Heparan Sulphate Proteoglycans in the Normal and Malignant Phenotype. Int. J. Mol. Sci..

[249] Melrose J. (2020). Perlecan, a modular instructive proteoglycan with diverse functional properties. Int. J. Biochem. Cell Biol..

[250] Loebel C., Mauck R.L., Burdick J.A. (2019). Local nascent protein deposition and remodelling guide mesenchymal stromal cell mechanosensing and fate in three-dimensional hydrogels. Nat. Mater..

[251] Loebel C., Kwon M.Y., Wang C., Han L., Mauck R.L., Burdick J.A. (2020). Metabolic Labeling to Probe the Spatiotemporal Accumulation of Matrix at the Chondrocyte-Hydrogel Interface. Adv. Funct. Mater..

[252] Zhou M., Lozano N., Wychowaniec J.K., Hodgkinson T., Richardson S.M., Kostarelos K., Hoyland J.A. (2019). Graphene oxide: A growth factor delivery carrier to enhance chondrogenic differentiation of human mesenchymal stem cells in 3D hydrogels. Acta Biomater..

[253] Ligorio C., Zhou M., Wychowaniec J.K., Zhu X., Bartlam C., Miller A.F., Vijayaraghavan A., Hoyland J.A., Saiani A. (2019). Graphene oxide containing self-assembling peptide hybrid hydrogels as a potential 3D injectable cell delivery platform for intervertebral disc repair applications. Acta Biomater..

[254] Vyas C., Ates G., Aslan E., Hart J., Huang B., Bartolo P. (2020). Three-Dimensional Printing and Electrospinning Dual-Scale Polycaprolactone Scaffolds with Low-Density and Oriented Fibers to Promote Cell Alignment. 3D Print. Addit. Manuf..

[255] Chang G., Kim H.J., Vunjak-Novakovic G., Kaplan D.L., Kandel R. (2010). Enhancing annulus fibrosus tissue formation in porous silk scaffolds. J. Biomed. Mater. Res. Part A.

[256] Kandel R., Santerre P., Massicotte E., Hurtig M., Shapiro I., Risbud M. (2014). Tissue engineering of the intervertebral disc. The Intervertebral Disc: Molecular and Structural Studies of the Disc in Health and Disease.

[257] Attia M., Santerre J.P., Kandel R.A. (2011). The response of annulus fibrosus cell to fibronectin-coated nanofibrous polyurethane-anionic dihydroxyoligomer scaffolds. Biomaterials.

[258] Pirvu T., Blanquer S.B.G., Benneker L.M., Grijpma D.W., Richards R.G., Alini M., Eglin D., Grad S., Li Z. (2015). A combined biomaterial and cellular approach for annulus fibrosus rupture repair. Biomaterials.

[259] Shamsah A.H., Cartmell S.H., Richardson S.M., Bosworth L.A. (2019). Mimicking the Annulus Fibrosus Using Electrospun Polyester Blended Scaffolds. Nanomaterials.

[260] Chong J.E., Santerre, Paul J., Kandel R.A., Mount R.A.K., Hospital S. (2020). Generation of an in vitro model of the outer annulus fibrosus-cartilage interface. JOR Spine.

[261] Turner K.G., Ahmed N., Santerre J.P., Kandel R.A. (2014). Modulation of annulus fibrosus cell alignment and function on oriented nanofibrous polyurethane scaffolds under tension. Spine J..

[262] Iu J., Santerre J.P., Kandel R.A. (2018). Towards engineering distinct multi-lamellated outer and inner annulus fibrosus tissues. J. Orthop. Res..

[263] Iu J., Santerre J.P., Kandel R.A. (2014). Inner and outer annulus fibrosus cells exhibit differentiated phenotypes and yield changes in extracellular matrix protein composition in vitro on a polycarbonate urethane scaffold. Tissue Eng. Part A.

[264] Liu C., Zhu C., Li J., Zhou P., Chen M., Yang H., Li B. (2015). The effect of the fibre orientation of electrospun scaffolds on the matrix production of rabbit annulus fibrosus-derived stem cells. Bone Res..

[265] Zhou P., Chu G., Yuan Z., Wang H., Zhang W., Mao Y., Zhu X., Chen W., Yang H., Li B. (2021). Regulation of differentiation of annulus fibrosus-derived stem cells using heterogeneous electrospun fibrous scaffolds. J. Orthop. Transl..

[266] Zhu C., Li J., Liu C., Zhou P., Yang H., Li B. (2016). Modulation of the gene expression of annulus fibrosus-derived stem cells using poly(ether carbonate urethane)urea scaffolds of tunable elasticity. Acta Biomater..

[267] Zhu C., Li J., Liu C., Zhou P., Yang H., Li B. (2015). Effect of scaffold elasticity on the gene expression of annulus fibrosus-derived stem cells. Data Brief.

[268] Chu G., Yuan Z., Zhu C., Zhou P., Wang H., Zhang W., Cai Y., Zhu X., Yang H., Li B. (2019). Substrate stiffness- and topography-dependent differentiation of annulus fibrosus-derived stem cells is regulated by Yes-associated protein. Acta Biomater..

[269] Vernengo A.J., Grad S., Eglin D., Alini M., Li Z. (2020). Bioprinting Tissue Analogues with Decellularized Extracellular Matrix Bioink for Regeneration and Tissue Models of Cartilage and Intervertebral Discs. Adv. Funct. Mater..

[270] Vadalà G., Mozetic P., Rainer A., Centola M., Loppini M., Trombetta M., Denaro V. (2012). Bioactive electrospun scaffold for annulus fibrosus repair and regeneration. Eur. Spine J..

[271] Thorvaldsson A., Silva-Correia J., Oliveira J.M., Reis R.L., Gatenholm P., Walkenström P. (2013). Development of nanofiber-reinforced hydrogel scaffolds for nucleus pulposus regeneration by a combination of electrospinning and spraying technique. J. Appl. Polym. Sci..

[272] Choy A.T.H., Chan B.P. (2015). A structurally and functionally biomimetic biphasic scaffold for intervertebral disc tissue engineering. PLoS ONE.

[273] Bowles R.D., Setton L.A. (2017). Biomaterials for intervertebral disc regeneration and repair. Biomaterials.

[274] Moriguchi Y., Borde B., Grunert P., Khair T., Hudson K., Alimi M., Bonassar L., Hartl R. (2015). Annular Repair Using High-Density Collagen Gels Seeded with Fibrochondrocytes: In Vivo Outcome in the Rodent Spine. Spine J..

[275] Moriguchi Y., Mojica-Santiago J., Grunert P., Pennicooke B., Berlin C., Khair T., Navarro-Ramirez R., Ricart Arbona R.J., Nguyen J., Härtl R. (2017). Total disc replacement using tissue-engineered intervertebral discs in the canine cervical spine. PLoS ONE.

[276] Murphy S.V., Atala A. (2014). 3D bioprinting of tissues and organs. Nat. Biotechnol..

[277] Groll J., Burdick J.A., Cho D.W., Derby B., Gelinsky M., Heilshorn S.C., Jüngst T., Malda J., Mironov V.A., Nakayama K. (2019). A definition of bioinks and their distinction from biomaterial inks. Biofabrication.

[278] Hospodiuk M., Dey M., Sosnoski D., Ozbolat I.T. (2017). The bioink: A comprehensive review on bioprintable materials. Biotechnol. Adv..

[279] Vanaei S., Parizi M.S., Vanaei S., Salemizadehparizi F., Vanaei H.R. (2021). An Overview on Materials and Techniques in 3D Bioprinting Toward Biomedical Application. Eng. Regen..

[280] Shiwarski D.J., Hudson A.R., Tashman J.W., Feinberg A.W. (2021). Emergence of FRESH 3D printing as a platform for advanced tissue biofabrication. APL Bioeng..

[281] Moxon S., Grover L.M., Smith A. (2016). Bioprinting multilayered hydrogels using 3D suspended manufacturing. Front. Bioeng. Biotechnol..

[282] Moxon S.R., Corbett N.J., Fisher K., Potjewyd G., Domingos M., Hooper N.M. (2019). Blended alginate/collagen hydrogels promote neurogenesis and neuronal maturation. Mater. Sci. Eng. C.

[283] Potjewyd G., Moxon S., Wang T., Domingos M., Hooper N.M. (2018). Tissue Engineering 3D Neurovascular Units: A Biomaterials and Bioprinting Perspective. Trends Biotechnol..

[284] Senior J.J., Cooke M.E., Grover L.M., Smith A.M. (2019). Fabrication of Complex Hydrogel Structures Using Suspended Layer Additive Manufacturing (SLAM). Adv. Funct. Mater..

[285] De Pieri A., Byerley A.M., Musumeci C.R., Salemizadehparizi F., Vanderhorst M.A., Wuertz-Kozak K. (2020). Electrospinning and 3D bioprinting for intervertebral disc tissue engineering. JOR Spine.

[286] Sun B., Lian M., Han Y., Mo X., Jiang W., Qiao Z., Dai K. (2021). A 3D-Bioprinted dual growth factor-releasing intervertebral disc scaffold induces nucleus pulposus and annulus fibrosus reconstruction. Bioact. Mater..

[287] Hu D., Wu D., Huang L., Jiao Y., Li L., Lu L., Zhou C. (2018). 3D bioprinting of cell-laden scaffolds for intervertebral disc regeneration. Mater. Lett..

[288] Zechuan Y., Chunde L.I., Haolin S. (2016). Research advances of three-dimension printing technology in vertebrae and intervertebral disc tissue engineering. Zhejiang Da Xue Xue Bao Yi Xue Ban.

[289] Zhang F., Liu J.T., Wang R., Lu T., Niu B.B., Qie J., Cai X., Zhang T., Ouyang P.R., He X.J. (2018). Establishment of the model of goat lumbar spinal fusion by 3D printing technology and experimental perioperative management. Zhongguo Gu Shang = China J. Orthop. Traumatol..

[290] Zhu M., Tan J., Liu L., Tian J., Li L., Luo B., Zhou C., Lu L. (2021). Construction of biomimetic artificial intervertebral disc scaffold via 3D printing and electrospinning. Mater. Sci. Eng. C.

[291] Oner T., Cengiz I.F., Pitikakis M., Cesario L., Parascandolo P., Vosilla L., Viano G., Oliveira J.M., Reis R.L., Silva-Correia J. (2017). 3D segmentation of intervertebral discs: From concept to the fabrication of patient-specific scaffolds. J. 3D Print. Med..

[292] Wu D., Tan J., Yao L., Tian J., Luo B., Li L., Zhou C., Lu L. (2021). Customized composite intervertebral disc scaffolds by integrated 3D bioprinting for therapeutic implantation. Compos. Part A Appl. Sci. Manuf..

[293] Rosenzweig D.H., Carelli E., Steffen T., Jarzem P., Haglund L. (2015). 3D-printed ABS and PLA scaffolds for cartilage and nucleus pulposustissue regeneration. Int. J. Mol. Sci..

[294] Cheng H.W., Luk K.D.K., Cheung K.M.C., Chan B.P. (2011). In vitro generation of an osteochondral interface from mesenchymal stem cell–collagen microspheres. Biomaterials.

[295] Chik T.K., Chooi W.H., Li Y.Y., Ho F.C., Cheng H.W., Choy T.H., Sze K.Y., Luk K.K.D., Cheung K.M.C., Chan B.P. (2015). Bioengineering a Multicomponent Spinal Motion Segment Construct—A 3D Model for Complex Tissue Engineering. Adv. Healthc. Mater..

[296] Afghah F., Dikyol C., Altunbek M., Koc B. (2019). Biomimicry in Bio-Manufacturing: Developments in Melt Electrospinning Writing Technology Towards Hybrid Biomanufacturing. Appl. Sci..

[297] de Ruijter M., Ribeiro A., Dokter I., Castilho M., Malda J. (2019). Simultaneous Micropatterning of Fibrous Meshes and Bioinks for the Fabrication of Living Tissue Constructs. Adv. Healthc. Mater..

[298] Hrynevich A., Haigh J.N., McMaster R., Youssef A., Blum C., Blunk T., Hochleitner G., Groll J., Dalton P.D. (2018). Dimension-Based Design of Melt Electrowritten Scaffolds. Small.

[299] Bas O., Lucarotti S., Angella D.D., Castro N.J., Meinert C., Wunner F.M., Rank E., Vozzi G., Klein T.J., Catelas I. (2018). Rational design and fabrication of multiphasic soft network composites for tissue engineering articular cartilage: A numerical model-based approach. Chem. Eng. J..

[300] Castilho M., Mouser V., Chen M., Malda J., Ito K. (2019). Bi-layered micro-fibre reinforced hydrogels for articular cartilage regeneration. Acta Biomater..

[301] Visser J., Melchels F.P.W., Jeon J.E., Van Bussel E.M., Kimpton L.S., Byrne H.M., Dhert W.J.A., Dalton P.D., Hutmacher D.W., Malda J. (2015). Reinforcement of hydrogels using three-dimensionally printed microfibres. Nat. Commun..

[302] Bas O., De-Juan-Pardo E.M., Meinert C., D’Angella D., Baldwin J.G., Bray L.J., Wellard R.M., Kollmannsberger S., Rank E., Werner C. (2017). Biofabricated soft network composites for cartilage tissue engineering. Biofabrication.

